# Strengthening of the C−F Bond in Fumaryl Fluoride with Superacids

**DOI:** 10.1002/chem.202104422

**Published:** 2022-02-10

**Authors:** Marie C. Bayer, Christoph Kremser, Christoph Jessen, Alexander Nitzer, Andreas J. Kornath

**Affiliations:** ^1^ Department Chemie Ludwig-Maximilians-Universität München Butenandtstrasse 5–13 (D) 81377 Munich Germany

**Keywords:** fluorides, quantum chemical calculations, +R effect, superacids, vibrational spectroscopy, X-ray structure analyses

## Abstract

The reaction of fumaryl fluoride with the superacidic solutions XF/MF_5_ (X=H, D; M=As, Sb) results in the formation of the monoprotonated and diprotonated species, dependent on the stoichiometric ratio of the Lewis acid to fumaryl fluoride. The salts [C_4_H_3_F_2_O_2_]^+^[MF_6_]^−^ (M=As, Sb) and [C_4_H_2_X_2_F_2_O_2_]^2+^([MF_6_]^−^)_2_ (X=H, D; M=As, Sb) are the first examples with a protonated acyl fluoride moiety. They were characterized by low‐temperature vibrational spectroscopy. Low‐temperature NMR spectroscopy and single‐crystal X‐ray structure analyses were carried out for [C_4_H_3_F_2_O_2_]^+^[SbF_6_]^−^ as well as for [C_4_H_4_F_2_O_2_]^2+^([MF_6_]^−^)_2_ (M=As, Sb). The experimental results are discussed together with quantum chemical calculations of the cations [C_4_H_4_F_2_O_2_ ⋅ 2 HF]^2+^ and [C_4_H_3_F_2_O_2_ ⋅ HF]^+^ at the B3LYP/aug‐cc‐pVTZ level of theory. In addition, electrostatic potential (ESP) maps combined with natural population analysis (NPA) charges were calculated in order to investigate the electron distribution and the charge‐related properties of the diprotonated species. The C−F bond lengths in the protonated dication are considerably reduced on account of the +R effect.

## Graphical abstract

**F plus**: The reaction of fumaryl fluoride with the superacids XF/MF_5_ (X=H, D; M=As, Sb) results in monoprotonated and diprotonated species: the first examples of a protonated acyl fluoride moiety. Surprisingly, the C−F bond lengths in the protonated cations are considerably shortened.

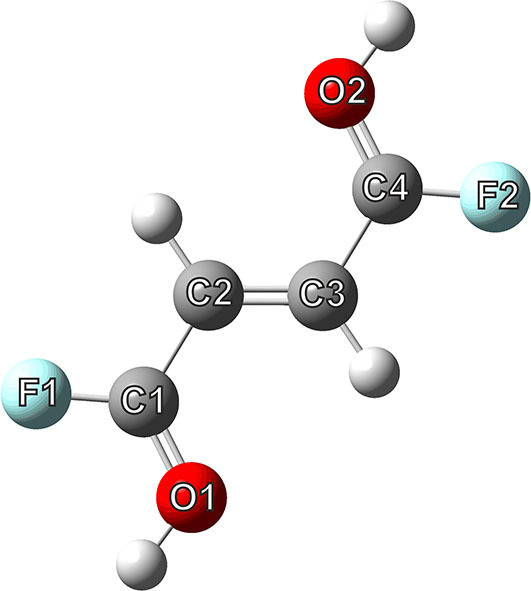

## Introduction

The prosperity of fluoroorganic compounds in every area of the chemical industry is conspicuous.^[1]^ The exceptional stability of the carbon‐fluorine bond is the most apparent property of organofluorine compounds.^[2]^ This stability, which is predicated by the electrostatic attraction between F^δ−^ and C^δ+^, distinguishes acyl fluorides from acyl halides in general.[^1^, ^3^] Acyl fluorides are key intermediates, serving as synthetic fragments, such as RCO and F sources, with an extensive range of applications in organic chemistry.[^3^, ^4^] A series of acyl fluorides were treated with various Lewis acids resulting in the formation of acyl cations (oxocarbenium ions).[^5^, ^6^] In addition, the complex formation of acyl fluorides with Lewis acid fluorides in SO_2_ClF was previously reported.[^6^, ^7^] Recently, the oxygen‐coordinated adducts of fumaryl fluoride, containing two acyl fluoride moieties, were characterized.^[8]^ Fumaric acid,[^9^, ^10^] as well as several carboxylic acids,[^11^, ^12^] were previously investigated in superacidic systems. In contrast, the acyl fluoride moiety has not been examined in superacids so far. This prompted us to investigate fumaryl fluoride in different binary superacid systems regarding its basicity.

## Results and Discussion

The superacid systems HF/MF_5_ (M=As, Sb) led to the formation of the monoprotonated and diprotonated fumaryl fluoride. An excess of the Lewis acids (AsF_5_ or SbF_5_) is necessary to synthesize the dication [C_4_H_4_F_2_O_2_]^2+^ according to Equation 1.

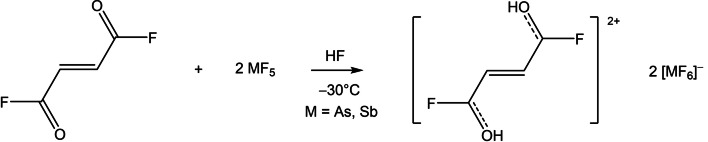




An equimolar amount of the Lewis acids in reference to fumaryl fluoride is required to form the salt containing the monoprotonated cation [Eq. 2].

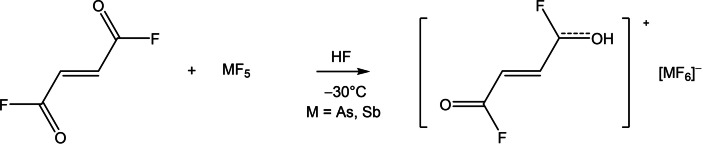




The reactions were carried out at a temperature of −30 °C. Excess of anhydrous hydrogen fluoride, which was used as both solvent and reagent, was removed at −78 °C in a dynamic vacuum. The salts [C_4_H_4_F_2_O_2_]^2+^([AsF_6_]^−^)_2_ (**1**) and [C_4_H_4_F_2_O_2_]^2+^([SbF_6_]^−^)_2_ (**2**) were obtained as colorless crystalline solids, which are stable up to 15 °C. The colorless crystals of [C_4_H_3_F_2_O_2_]^+^[AsF_6_]^−^ (**3**) and [C_4_H_3_F_2_O_2_]^+^[SbF_6_]^−^ (**4**) decompose above −10 °C. The corresponding deuterated species [C_4_H_2_D_2_F_2_O_2_]^2+^([AsF_6_]^−^)_2_ (**5**) and [C_4_H_2_D_2_F_2_O_2_]^2+^([SbF_6_]^−^)_2_ **(6)** were prepared by modifying the superacid systems to DF/MF_5_ (M=As, Sb).

To test whether the protonation is possible under milder conditions, the superacidic solutions HF/MF_4_ (M=Ge, S) and HF/BF_3_, which are lower in acidity than fluoroantimonic acid,^[13]^ were used. However, no reaction was observable.

### Crystal structure of [C_4_H_4_F_2_O_2_]^2+^([AsF_6_]^−^)_2_

**Figure 1:**
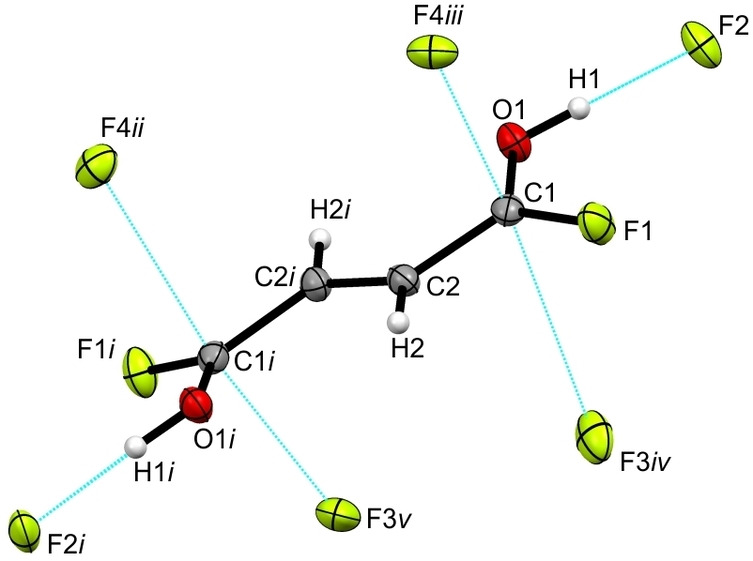
Projection of the cation in [C_4_H_4_F_2_O_2_]^2+^([AsF_6_]^−^)_2_ and its interatomic contacts (displacement ellipsoids with 50 % probability). Symmetry operations: *i*=1−*x*, *y*, 0.5−*z*; *ii*=*x*, 1−*y*, −0.5+*z*; *iii*=1−*x*, 1−*y*, 1−*z*; *iv*=1−*x*, −*y*, 1−*z*; *v*=*x*, −*y*, −0.5+*z*. Interatomic contacts are drawn as dashed blue lines.

[C_4_H_4_F_2_O_2_]^2+^([AsF_6_]^−^)_2_ (**1**) crystallizes in the orthorhombic space group *Pbcn* with four formula units per unit cell. Figure S1 in the Supporting Information shows the formula unit of **1** and Table S1 summarizes selected structural parameters. The cation in the crystal structure of **1**, which is shown in Figure 1, exists as the *cis‐cis* conformer. Selected bond lengths and angles of *cis‐cis*‐fumaryl fluoride^[8]^ compared with **1** are summarized in Table 1. The diprotonation of fumaryl fluoride has a great influence on the C=O bond lengths. The C1−O1 bonds are with 1.223(2) Å significantly elongated compared to fumaryl fluoride,^[8]^ and are in the range between formal C−O single (1.43 Å) and C=O double bonds (1.19 Å).^[14]^ In contrast, the C1−F1 bonds are with 1.281(2) Å shorter than formal C−F single bonds (1.36 Å).^[14]^ In comparison with the reactant^[8]^ the C1−F1 bonds are significantly shortened. The C=C bond is also affected by the diprotonation. By contrast with fumaryl fluoride,^[8]^ the C2−C2*i* is with 1.330(3) Å significantly elongated and agrees well with formal C=C bond lengths (1.33 Å).^[14]^ The C1−C2 single bonds are not significantly affected by the diprotonation. The most pronounced effect of the diprotonation on the bond angles is for the O−C−C and F−C−C angles. The O1−C1−C2 angle is reduced by 3.9° to 123.4(2)°, whereas the F1−C1−C2 bond angle is widened to 116.4(2)°. The protonated acyl fluoride groups are twisted against one another by 10° out of the carbon‐skeleton plane, resulting in a slightly distorted planar structure.

**Table 1:** Comparison of selected bond lengths and angles of *cis,cis‐* and *trans,trans‐*fumaryl fluoride,^[8]^ [C_4_H_4_F_2_O_2_]^2+^([AsF_6_]^−^)_2_ (**1**) (symmetry operation: *i*=1−*x*, *y*, 0.5−*z*), [C_4_H_4_F_2_O_2_]^2+^([SbF_6_]^−^)_2_ (**2**) (symmetry operation: *i*=2−*x*, −*y*, 1−*z*.) and [C_4_H_3_F_2_O_2_]^+^[SbF_6_]^−^ (**4**) with estimated standard deviation in parentheses.

	*cis‐cis*‐C_4_H_2_F_2_O_2_ ^[8]^	*trans‐trans*‐C_4_H_2_F_2_O_2_ ^[8]^		**1**	**2**	**4**
Bond length [Å]						
C=C	1.314(2)	1.322(6)	C2−C2*i*	1.330(3)	1.336(5)	
			C2−C3			1.334(7)
C−C	1.477(2)	1.473(4)	C1−C2	1.465(2)	1.461(4)	1.476(8)
			C3−C4			1.454(7)
C=O	1.190(2)	1.177(4)	C1−O1	1.223(2)	1.223(4)	1.187(6)
			C4−O2			1.239(6)
C−F	1.334(2)	1.349(3)	C1−F1	1.281(2)	1.287(4)	1.332(6)
			C4−F2			1.285(6)
Bond angle [°]						
O−C−F	120.2(1)	119.5(3)	O1−C1−F1	120.2(2)	120.6(3)	120.7(5)
			O2−C4−F2			118.6(4)
O−C−C	127.3(2)	127.2(3)	O1−C1−C2	123.4(2)	121.1(3)	126.4(5)
			O2−C4−C3			122.5(5)
F−C−C	112.5(1)	113.2(3)	F1−C1−C2	116.4(2)	118.2(3)	112.9(4)
			F2−C4−C3			118.9(4)
C−C−C	120.3(2)	123.1(3)	C1−C2−C2*i*	119.1(2)	120.1(3)	
			C1−C2−C3			122.1(5)
			C4−C3−C2			118.6(5)

The As−F bond lengths in the [AsF_6_]^−^ anion are in the range between 1.702(1) and 1.717(1) Å. These values are in accordance with reported As−F bond lengths of hexafluoroarsenate anions.^[15]^ The As1−F2 bond (1.800(1) Å) is involved in hydrogen bonding in the crystal packing and is therefore elongated in comparison with the other As−F bonds. Besides, the bond angles deviate up to 5° from the ideal octahedron angles, indicating a distorted structure of the anion. In the crystal packing of **1**, the cation is connected with the anions by different types of interatomic contacts. A cutout of these contacts is given in Figure 1, and the detailed illustration is shown in Figure S2.

The hydrogen bonds O1−H1⋅⋅⋅F2 with donor‐acceptor distances of 2.429(2) Å are categorized as strong hydrogen bonds according to the classification of Jeffrey.^[16]^ Additionally, two interatomic contacts between C1 and F3*iv* (2.868(2) Å) and between C1 and F4*iii* (2.614(2) Å) are observed. Both interatomic C−F distances are below the sum of the van der Waals radii (3.17 Å).^[17]^ A zigzag chain structure is formed through the O1−H1⋅⋅⋅F2 hydrogen bonds and the C1⋅⋅⋅F4*iii* contacts along the *c*‐axis, as it is shown in Figure S3.

### Crystal structure of [C_4_H_4_F_2_O_2_]^2+^([SbF_6_]^−^)_2_

**Figure 2:**
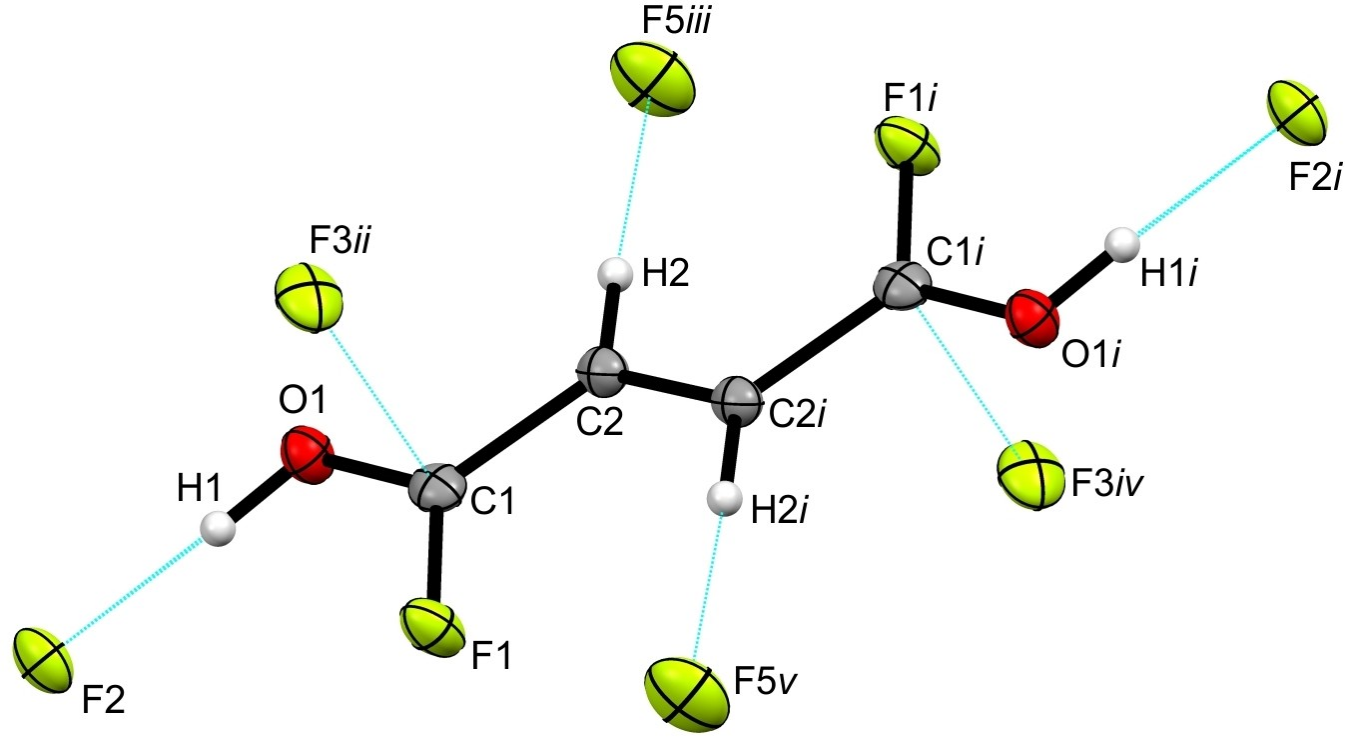
Visualization of the cation in [C_4_H_4_F_2_O_2_]^2+^([SbF_6_]^−^)_2_ with its interatomic contacts (displacement ellipsoids with 50 % probability). Symmetry operations: *i*=2−*x*, −*y*, 1−*z*; *ii*=2−*x*, 1−*y*, 1−*z*; *iii*=1+x, −1+*y*, *z*; *iv*=*x*, −1+*y*, *z*; *v*=1−*x*, 1−*y*, 1−*z*. Interatomic contacts are shown as dashed blue lines.

[C_4_H_4_F_2_O_2_]^2+^([SbF_6_]^−^)_2_ (**2**) crystallizes in the monoclinic space group *P*2_1_/*c* with two formula units per unit cell. Figure S4 shows the formula unit of **2** and selected structural parameters are listed in Table S2. The crystal structure of **1** and **2** differ in terms of their cationic conformational structure. As the cation of **1** is a *cis‐cis* conformer, the cation in the crystal structure of **2**, depicted in Figure 2, exists as the *trans‐trans* conformer.

Selected bond lengths and angles of *cis‐cis‐* and *trans‐trans‐*fumaryl fluoride,^[8]^ **1** and **2** are listed in Table 1. By analogy with **1**, the diprotonation of fumaryl fluoride causes a significant elongation of the C1−O1 bond (1.223(4) Å), whereas the C1−F1 bond is significantly shortened (1.287(4) Å), compared to the starting material.^[8]^ No significant difference in the lengths of the double C=C and single C−C bonds is observable in comparison to the neutral *trans‐trans‐*compound.^[8]^ The bond lengths of the cations in the crystal structure of **1** and **2** are consistent with one another. The bond angle O1−C1−C2 is significantly reduced to 121.1(3)° compared to **1** and *trans‐trans‐*fumaryl fluoride.^[8]^ Though, the bond angle F1−C1−C2 is significantly widened to 118.2(3)° in contrast to **1** and the neutral compound.^[8]^ The protonated acyl fluoride groups are twisted against one another by 6° out of the carbon‐skeleton plane, leading to a slightly distorted planar structure.

The Sb−F bond lengths of the [SbF_6_]^−^ anion range between 1.858(2) and 1.934(2) Å, revealing a distorted octahedral structure. The values are in accordance with literature data for [SbF_6_]^−^ anions.[^18^, ^19^]

In the solid state of **2** three different interatomic contacts connect the cations and the anions, illustrated in detail in Figure S5. Figure 2 shows a cutout of the interatomic contacts. The O1−H1⋅⋅⋅F2 hydrogen bond (2.426(3) Å), categorized as strong,^[16]^ agrees well with the corresponding hydrogen bond in the crystal structure of **1**. On the other hand, the C2−H2⋅⋅⋅F5*iii* hydrogen bond with a donor‐acceptor distance of 3.072(4) Å is classified as moderate.^[16]^ Additionally, one interatomic contact between C1 and F3*ii* (2.578(4) Å) is detected, which is below the sum of the van der Waals radii of 3.17 Å.^[17]^

### Crystal structure of [C_4_H_3_F_2_O_2_]^+^[SbF_6_]^−^

**Figure 3:**
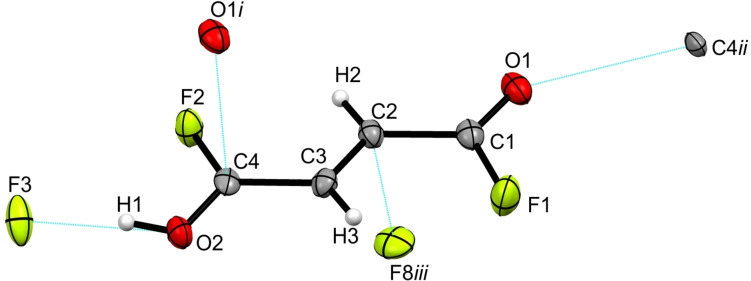
Illustration of the cation in [C_4_H_3_F_2_O_2_]^+^[SbF_6_]^−^ and its interatomic contacts (displacement ellipsoids with 50 % probability). Symmetry operations: *i*=0.5+*x*, 1.5−*y*, −0.5+*z*; *ii*=−0.5+*x*, 1.5−*y*, 0.5+*z*; *iii*=−1+*x*, 1−*y*, 0.5+*z*. Interatomic contacts are shown as dashed blue lines.

[C_4_H_3_F_2_O_2_]^+^[SbF_6_]^−^ (**4**) crystallizes in the monoclinic space group *Cc* with four formula units per unit cell. Figure S6 shows the asymmetric unit of **4** and selected structural parameters are summarized in Table S3. In compliance with the crystal structure of **2**, the cation of **4**, which is illustrated in Figure 3, exists as the *trans‐trans* conformer.

In order to better compare the structural data of the monoprotonated cation, selected bond lengths and angles of *cis‐cis‐* and *trans‐trans‐*fumaryl fluoride,^[8]^ as well as **1**, **2** and **4** are summarized in Table 1. The C=O bond lengths of the monoprotonated cation differ considerably from each other. Since the unprotonated C1−O1 bond (1.187(6) Å) remains unaffected and conforms to the *trans‐trans*‐fumaryl fluoride,^[8]^ the protonated C4−O2 bond is with 1.239(6) Å significantly elongated and accords with the diprotonated species of **2**. The C−F bonds in **4** vary in bond distances as well. The C1−F1 bond length (1.332(6) Å) of the unprotonated carbonyl fluoride group remains unchanged and corresponds to *trans‐trans‐*fumaryl fluoride.^[8]^ However, the C4−F2 bond distance is with 1.285(6) Å significantly shortened compared to the reactant,^[8]^ but in good agreement with the diprotonated cation of **2**. The C=C double bond and the C−C single bonds are in accordance with the starting material,^[8]^ as well as with **2**. The O2−C4−C3 angle is reduced to 122.5(5)° due to the protonation, whereas the unprotonated O1−C1−C2 bond angle remains constant and agrees well with the *trans‐trans* conformer.^[8]^ Since the F2−C4−C3 bond angle is significantly widened to 118.9(4)° based on the monoprotonation, which conforms to **2**, the F1−C1−C2 bond angle is unaltered and in accordance with the reactant.^[8]^ A slightly distorted planar structure of the monocation results from the acyl groups being twisted against each other by around 8° out of the carbon‐skeleton plane.

In the [SbF_6_]^−^ anion the Sb−F bond distances range between 1.863(3) and 1.873(3) Å, and are in the typical range of Sb−F bond lengths.[^18^, ^20^] The Sb1−F3 bond (1.936(3) Å) is elongated in comparison to reported Sb−F bond distances, owing to the fact that it is involved in hydrogen bonding in the crystal structure of **4**. A distorted octahedral structure of the [SbF_6_]^−^ is implied by the bond angles, which deviate by up to about 5° from the ideal octahedron angles.

The ions are linked together by three different types of interatomic contacts in the crystal structure of **4**, which are visualized elaborately in Figure S7. Figure 3 depicts a detail of the interatomic contacts. Referred to the categorization of Jeffrey^[16]^ the hydrogen bond O2−H1⋅⋅⋅F3 (2.470(5) Å), connecting the cations with the anions, is designated as strong. Additionally the interatomic contact between C2 and F8*iii* (2.839(7) Å) connects the cations with the anions, which is below the sum of the van der Waals radii (3.17 Å).^[17]^ A cation‐cation contact is observed between C4 and O1*i* forming a zigzag chain along the *c*‐axis, which is illustrated in Figure S8. This C4⋅⋅⋅O1*i* distance of 2.809(6) Å is below the sum of the van der Waals radii (3.22 Å).^[17]^

### Vibrational spectra of [C_4_H_4_F_2_O_2_]^2+^([MF_6_]^−^)_2_ (M=As, Sb)

**Figure 4:**
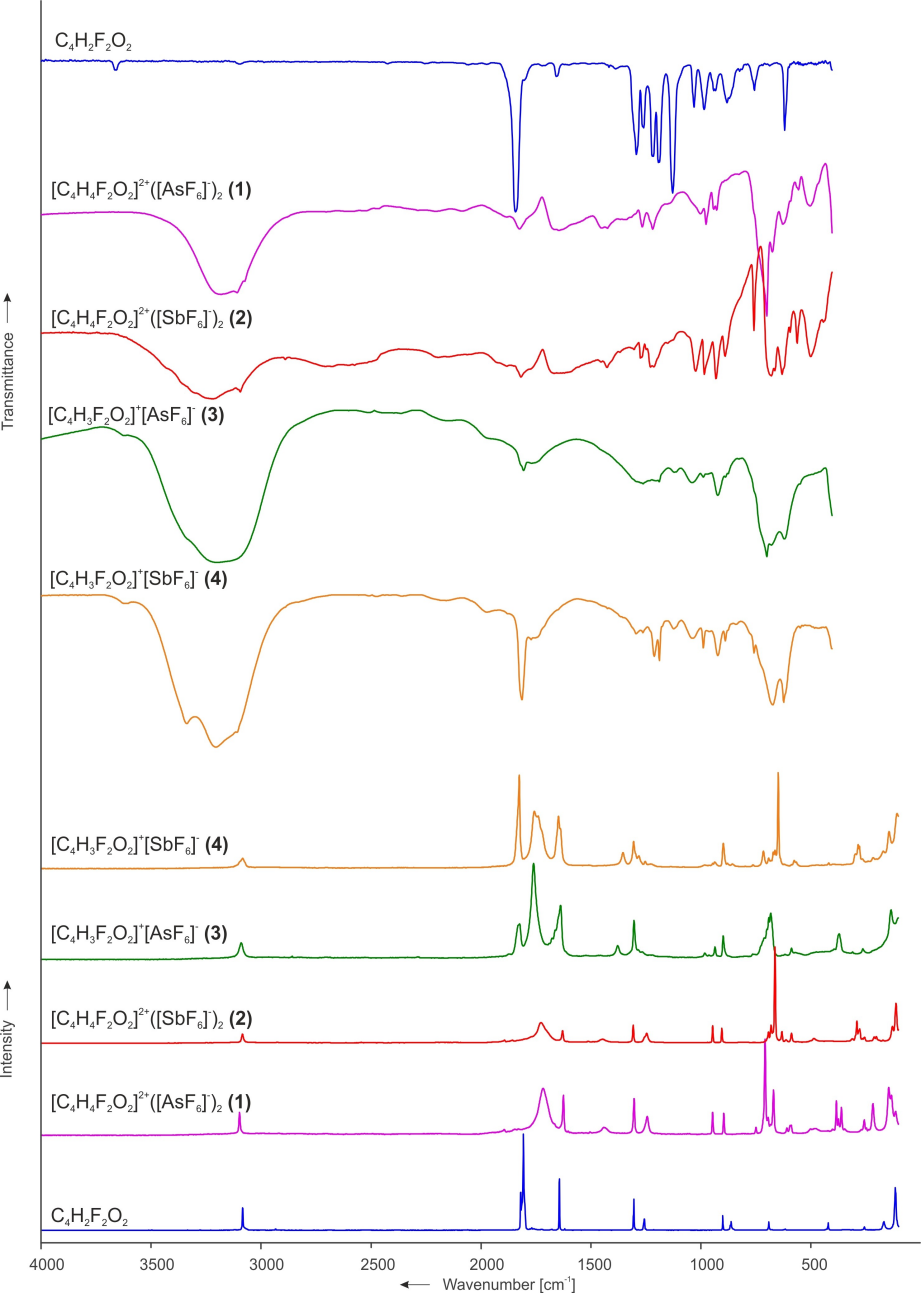
Low‐temperature Raman and IR spectra of crystalline fumaryl fluoride,^[8]^ [C_4_H_4_F_2_O_2_]^2+^([AsF_6_]^−^)_2_ (**1**), [C_4_H_4_F_2_O_2_]^2+^([SbF_6_]^−^)_2_ (**2**), [C_4_H_3_F_2_O_2_]^+^[AsF_6_]^−^ (**3**) and [C_4_H_3_F_2_O_2_]^+^[SbF_6_]^−^ (**4**).

Figure 4 shows the low‐temperature vibrational spectra of [C_4_H_4_F_2_O_2_]^2+^([AsF_6_]^−^)_2_ (**1**), [C_4_H_4_F_2_O_2_]^2+^([SbF_6_]^−^)_2_ (**2**) and crystalline fumaryl fluoride.^[8]^ The vibrational spectra of the deuterium isotopomeric salts [C_4_H_2_D_2_F_2_O_2_]^2+^([AsF_6_]^−^)_2_ (**5**) and [C_4_H_2_D_2_F_2_O_2_]^2+^([SbF_6_]^−^)_2_ (**6**) are illustrated in Figure S9 (Supporting Information). Selected experimental vibrational frequencies of **1** and **2** as well as the quantum chemically calculated frequencies of the cation [C_4_H_4_F_2_O_2_ ⋅ 2 HF]^2+^ are summarized in Table 2. The complete data are given in Table S4. The experimental vibrational frequencies of **5** and **6** accompanied by the calculated frequencies of the cation [C_4_H_2_D_2_F_2_O_2_ ⋅ 2 HF]^2+^ are given in Table S5.

**Table 2:** Selected experimental vibrational frequencies [cm^−1^] of [C_4_H_4_F_2_O_2_]^2+^([MF_6_]^−^)_2_ (M=As, Sb) and calculated vibrational frequencies [cm^−1^] of [C_4_H_4_F_2_O_2_ ⋅ 2 HF]^2+^.

[C_4_H_4_F_2_O_2_]^2+^([AsF_6_]^−^)_2_ exp.^[a]^		[C_4_H_4_F_2_O_2_]^2+^([SbF_6_]^−^)_2_ exp.^[a]^		[C_4_H_4_F_2_O_2_ ⋅ 2 HF]^2+^ calcd.^[b]^	Assignment		
IR	Raman	IR	Raman	IR/Raman			
3109 vs		3094 vs		3190 (60/0)	*ν*_21_	*B_u_*	*ν*_as_(C−H)
	3098 (24)		3085 (9)	3188 (0/79)	*ν*_1_	*A_g_*	*ν*_s_(C−H)
3182 vs		3217 vs		2490 (7585/0)	*ν*_22_	*B_u_*	*ν*_as_(O−H)
	1717 (49)		1726 (21)	1713 (0/8)	*ν*_3_	*A_g_*	*ν*_s_(C=O)
	1625 (42)		1629 (13)	1685 (0/384)	*ν*_4_	*A_g_*	*ν*(C=C)
1641 m		1666 vs		1681 (816/0)	*ν*_23_	*B_u_*	*ν*_as_(C=O)
1450 m		1452 s		1485 (717/0)	*ν*_24_	*B_u_*	*v*_as_(C−F)
	1441 (8)		1445 (4)	1454 (0/4)	*ν*_5_	*A_g_*	*v*_s_(C−F)
	1244 (20)	1246 s	1246 (10)	1280 (0/38)	*ν*_7_	*A_g_*	*δ*_s_(COH)
	946 (24)		946 (18)	995 (0/6)	*ν*_8_	*A_g_*	*ν*_s_(C−C)
926 w		930 vs		933 (127/0)	*ν*_27_	*B_u_*	*ν*_as_(C−C)
	693 (19)		692 (12)	712 (0/11)	*ν*_9_	*A_g_*	*δ*_s_(COF)

[a] Abbrevations for IR intensities: v=very, s=strong, m=medium, w=weak. IR intensities in km/mol; Raman intensities in Å^4^/u. Experimental Raman activities are relative to a scale of 1 to 100. [b] Calculated at the B3LYP/aug‐cc‐pVTZ level of theory.

As crystalline fumaryl fluoride consists mainly of the *cis‐cis* conformers,^[8]^ we assume the same conformational arrangement for the [C_4_H_4_F_2_O_2_]^2+^ cation, which is confirmed by the single‐crystal X‐ray structural analysis of **1**. Therefore the following calculated vibrational frequencies refer to the *cis,cis* conformer of the [C_4_H_4_F_2_O_2_]^2+^ cation, displaying *C*_2h_ symmetry with 30 fundamental vibrations (11*A_g_* +4*B_g_* +5*A_u_* +10*B_u_*). The rule of mutual exclusion applies by reason of the inversion center.^[21]^ The assignment of the vibrational modes was performed by analyzing the Cartesian displacement vectors of the calculated vibrational modes of [C_4_H_4_F_2_O_2_ ⋅ 2 HF]^2+^ and by comparing to literature data of fumaryl fluoride.[^8^, ^22^] To improve consistency of the calculated frequencies with the experimental ones, two HF molecules were attached to the gas phase structure of the cation simulating hydrogen bonds in the solid state.^[23]^

The antisymmetric O−H stretching vibration, indicating the successful protonation, is detected in the IR spectra at 3182 (**1**) and 3217 cm^−1^ (**2**). However, the corresponding calculated value for the O−H stretching mode is underestimated. The two added HF molecules, simulating hydrogen bonds in the crystal structure, result in the calculation of lower values for the O−H stretching vibration.^[23]^ We assume the hydrogen bonds being weaker than supposed in the calculation. Owing to the poor polarizability of the O−H bond, corresponding Raman lines are not observable. By contrast, the O−D stretching vibrations are identifiable in the Raman spectra at 2307 (**5**) and 2293 cm^−1^ (**6**). In the IR spectra the antisymmetric O−D stretching modes are observed at 2359 (**5**) and 2318 cm^−1^ (**6**). These H/D red‐shifts are consistent with the Teller‐Redlich rule for a H/D isotopic effect.^[21]^ The C−H stretching vibrations of **1** are slightly blue‐shifted by 14 cm^−1^ (3098 cm^−1^ (Ra)) and by 12 cm^−1^ (3109 cm^−1^ (IR)) by comparison with the starting material.^[8]^ Though, the C−H stretching modes of **2** remain unshifted. As a result of the twofold protonation the C=O bonds are weakened occurring in the Raman spectra at 1717 (**1**) and at 1726 cm^−1^ (**2**), and in the IR spectra at 1641 (**1**) and at 1666 cm^−1^ (**2**). Compared to fumaryl fluoride,^[8]^ those vibrations are red‐shifted by up to 165 cm^−1^. The weakening of the C=O bonds is also observed in the crystal structure of **1** and **2**. The C=C stretching mode appears in the Raman spectra at 1625 (**1**) and at 1629 cm^−1^ (**2**), and by comparing to the neutral compound,^[8]^ it is slightly red‐shifted by 18 (**1**) and by 14 cm^−1^ (**2**). This indicates a slight elongation of the C=C double bond that is verified by the crystal structure of **1**. In the Raman spectra, the symmetric C−F stretching vibration is observed at 1441 (**1**) and at 1445 cm^−1^ (**2**). In contrast to fumaryl fluoride,^[8]^ it is blue‐shifted by 182 (**1**) and by 186 cm^−1^ (**2**), displaying a strengthening of the C−F bond as a result of the protonation, which is also observed in the crystal structure of **1** and **2**. Three Raman lines and two IR bands are expected for the anions [AsF_6_]^−^ and [SbF_6_]^−^ with ideal *O*_h_ symmetry. However, more vibrations are observed in the spectra of **1**, **2**, and **5**, **6**, signifying a distorted octahedral structure of the anions, confirmed by single‐crystal X‐ray structural analysis of **1** and **2**.

### Vibrational spectra of [C_4_H_3_F_2_O_2_]^+^[MF_6_]^−^ (M=As, Sb)

The low‐temperature vibrational spectra of [C_4_H_3_F_2_O_2_]^+^[AsF_6_]^−^ (**3**) and [C_4_H_3_F_2_O_2_]^+^[SbF_6_]^−^ (**4**) in comparison with the diprotonated salts and crystalline fumaryl fluoride^[8]^ are depicted in Figure 4. Table 3 combines selected experimental vibrational frequencies of **3**, **4** and the quantum chemically calculated frequencies of the cation [C_4_H_3_F_2_O_2_ ⋅ HF]^+^. The complete data can be found in Table S6.

**Table 3:** Selected experimental vibrational frequencies [cm^−1^] of [C_4_H_3_F_2_O_2_]^+^[MF_6_]^−^ (M=As, Sb) and calculated vibrational frequencies [cm^−1^] of [C_4_H_3_F_2_O_2_ ⋅ HF]^+^.

[C_4_H_3_F_2_O_2_]^+^[AsF_6_]^−^ exp.^[a]^		[C_4_H_3_F_2_O_2_]^+^[SbF_6_]^−^ exp.^[a]^		[C_4_H_3_F_2_O_2_ ⋅ HF]^+^ calcd.^[b]^	Assignment		
IR	Raman	IR	Raman	IR/Raman			
		3109 vs		3212 (26/29)	*ν*_1_	*A‘*	*ν*(C−H)^[c]^
	3090 (17)		3084 (11)	3199 (6/54)	*ν*_2_	*A‘*	*ν*(C−H)
3196 vs		3203 vs		3061 (2649/166)	*ν*_3_	*A‘*	*ν*(O−H)^[c]^
1803 m	1823 (37)	1813 s	1825 (97)	1872 (180/260)	*ν*_4_	*A‘*	*ν*(C=O)
	1637 (56)		1647 (54)	1675 (636/227)	*ν*_5_	*A‘*	*ν*(C=C)
1770 m	1761 (100)	1770 w	1757 (60)	1621 (181/2)	*ν*_6_	*A‘*	*ν*(C=O)^[c]^
	1378 (14)		1354 (17)	1489 (195/12)	*ν*_7_	*A‘*	*ν*(C−F)^[c]^
	1304 (41)		1305 (28)	1316 (7/29)	*ν*_8_	*A‘*	*δ*(CCH)
1290 m	1284 (10)	1292 w	1281 (13)	1280 (22/1)	*ν*_9_	*A‘*	*δ*(CCH)
1261 m	1265 (8)	1261 w	1252 (8)	1259 (205/24)	*ν*_10_	*A‘*	*δ*(COH)^[c]^
1205 m		1209 m	1227 (5)	1213 (433/9)	*ν*_11_	*A‘*	*ν*(C−F)
987 w	981 (7)	987 w	982 (4)	956 (27/12)	*ν*_12_	*A‘*	*ν*(C−C)^[c]^
922 m	935 (13)	920 m	936 (7)	916 (122/0)	*ν*_22_	*A‘‘*	*γ*(COH)^[c]^
885 w	899 (24)	887 w	898 (26)	882 (34/13)	*ν*_13_	*A‘*	*ν*(C−C)

[a] Abbrevations for IR intensities: v=very, s=strong, m=medium, w=weak. IR intensities in km/mol; Raman intensities in Å^4^/u. Experimental Raman activities are relative to a scale of 1 to 100. [b] Calculated at the B3LYP/aug‐cc‐pVTZ level of theory. [c] Protonated acyl fluoride moiety.

The monoprotonated cations reveal a *trans‐trans* conformational structure in the solid state, as described in the section above. The following calculated vibrational frequencies accordingly correspond to the *trans‐trans* conformer of the [C_4_H_3_F_2_O_2_]^+^ cation, having *C*_s_ symmetry with 27 fundamental vibrations (19 *A′*+8 *A′′*). The vibrational frequencies were assigned by examining the Cartesian displacement vectors of the calculated vibrational modes of [C_4_H_3_F_2_O_2_ ⋅ HF]^+^ as well as by comparing to literature data of fumaryl fluoride.[^8^, ^22^] The successful monoprotonation of fumaryl fluoride is shown, inter alia, in the O−H stretching vibration noted in the IR spectra at 3196 (**3**) and 3203 cm^−1^ (**4**). Admittedly the related calculated frequency for the *ν*(O−H) mode is underestimated, caused by the added HF molecule. The C−H stretching vibration of the monoprotonated cation is observed in the Raman spectra at 3090 cm^−1^ **3**, which is slightly blue‐shifted by 6 cm^−1^, and at 3084 cm^−1^ **4**, which remains unaltered compared to the neutral compound.^[8]^ Further evidence for the monoprotonation is the C=O stretching vibration. The vibration of the unprotonated C=O bond in the Raman spectra of **3** and **4** remains unshifted and agrees with that of the starting material.^[8]^ The stretching mode of the protonated C=O bond is red‐shifted in the Raman spectra by 58 cm^−1^ (1761 cm^−1^, **3**) and by 62 cm^−1^ (1757 cm^−1^, **4**), respectively, as well as in the IR spectra by 70 cm^−1^ (1770 cm^−1^ **3**, **4**) in comparison with the starting material.^[8]^ The weakening of the protonated C=O bond is in agreement with the crystal structure of **4**. The C=C stretching mode appearing in the Raman spectra at 1637 (**3**) and 1647 cm^−1^ (**4**), respectively, is comparable with that of the reactant^[8]^ and is hardly affected by the monoprotonation, which is in agreement with the crystal structure of **4**. In the Raman spectra, the C−F stretching vibration of the protonated carbonyl fluoride group is observed at 1378^1^ (**3**) and at 1354 cm^−1^ (**4**), respectively. In contrast to the *trans‐trans* conformer of the neutral compound,^[8]^ it is blue‐shifted by 121 (**3**) and by 97 cm^−1^ (**4**), respectively. The strengthening of this C−F bond length is in accordance with the observed shortening of the C4−F2 bond distance in the crystal structure of **4**. The C−F stretching vibration of the unprotonated group is also shifted to higher wavenumbers, appearing in the IR spectra at 1205 (**3**) and at 1209 cm^−1^ (**4**), respectively. For the octahedral anions [MF_6_]^−^ (M=As, Sb) five vibrational modes are estimated. Indeed more lines and bands are noticed in the spectra of **3** and **4**, thus implying a lowered symmetry of the structure of the anions, which agrees well with the crystal structure of **4**.

### NMR spectroscopy

^1^H, ^13^C and ^19^F NMR spectra of fumaryl fluoride dissolved in CDCl_3_ were recorded at 26 °C. A complete analysis of these spectra, including the ^13^C satellites, result in the NMR parameters that are shown in Table S7. Anhydrous hydrogen fluoride solutions of C_4_H_2_F_2_O_2_, as well as of the salts **1**, **2** and **4** were measured at −40 °C. Selected observed chemical shifts and spin‐spin coupling constants of **1**, **2** and **4** together with C_4_H_2_F_2_O_2_ in the different solvents are summarized in Table S8. Figure S10 illustrates the stacked ^1^H NMR spectra of C_4_H_2_F_2_O_2_ dissolved in CDCl_3_ and in *a*HF, respectively, and **1**, **2**, **4** in *a*HF. All of the experimental NMR spectra are depicted in Figures S11–S27.

**Figure 5:**
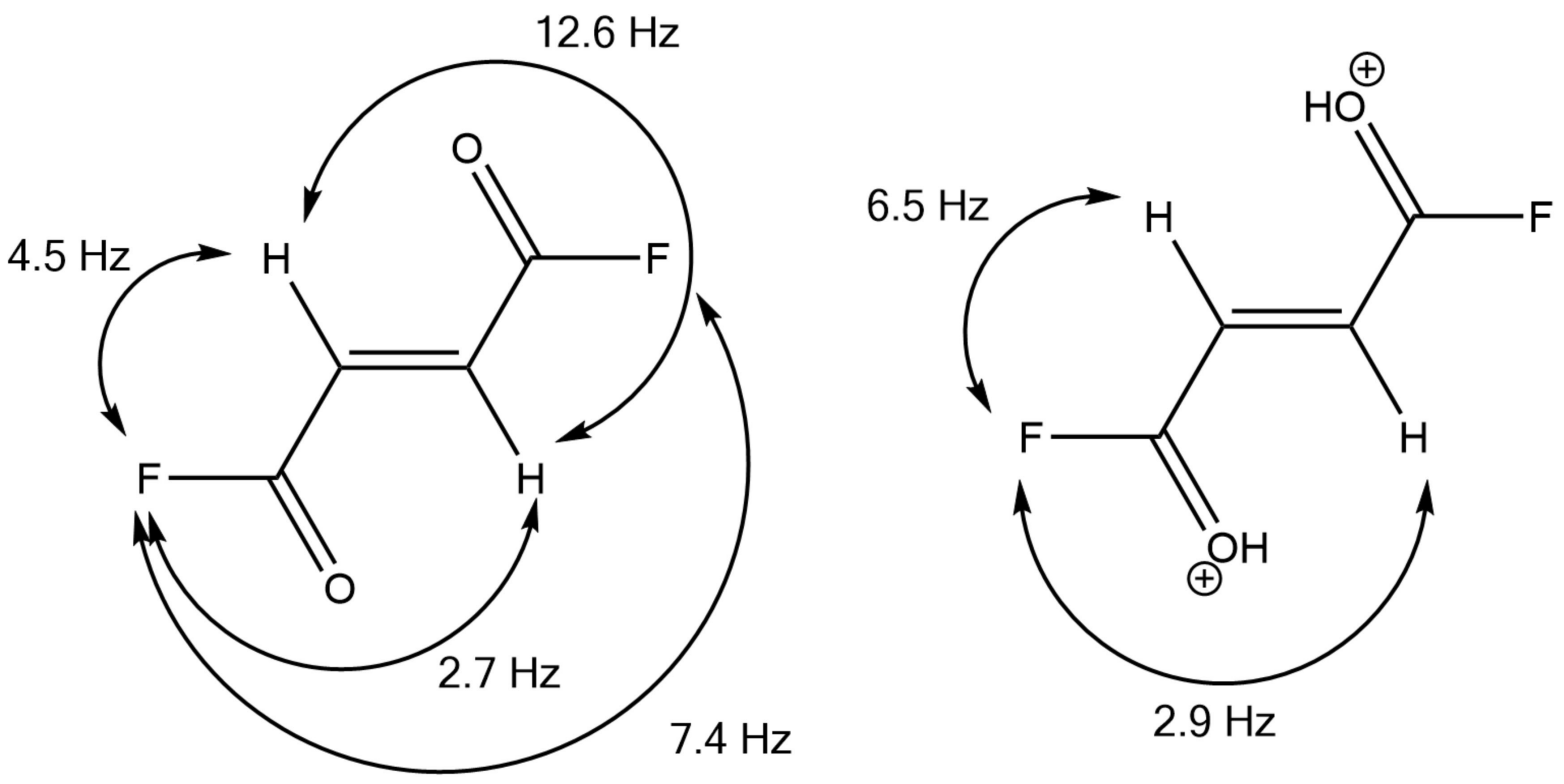
Molecular structure of fumaryl fluoride showing the ^1^H,^1^H, ^1^H,^19^F and ^19^F,^19^F *J* couplings (left). Cationic structure of [C_4_H_4_F_2_O_2_]^2+^ displaying the ^1^H,^19^F *J* couplings (right).

The NMR parameters of fumaryl fluoride in CDCl_3_ are consistent with those published in the literature.^[24]^ Depending on the solvent used the signals occur at different resonant frequencies. Fumaryl fluoride contains each two chemically equivalent protons and fluorine nuclei. Hence the ^1^H and ^19^F spectra of the four‐spin system is of the type AA′XX′.^[24]^ Figure 5 shows the molecular structure of fumaryl fluoride together with the spin–spin coupling constants. The higher electronegativity of the fluorine substituents results in a decreased ^1^H,^1^H *J* coupling of ^3^ *J*_HH_=12.6 Hz, compared to that of fumaric acid (^3^ *J*_HH_=15.7 Hz).^[25]^ The sp^2^ hybridization of the olefinic carbon atoms is confirmed by the ^1^H,^13^C *J* coupling of ^1^ *J*_CH_=175.5 Hz, which is increased compared to that of ethylene (^1^ *J*_CH_=156 Hz)^[26]^ due to the fluorine substituents.^[27]^

The ^1^H NMR spectra of **1**, **2** and **4** in *a*HF show each a signal at approximately 10 ppm, which is attributed to the C(OH)F moiety and confirms the successful protonation. A doublet of doublets at around 7.2 ppm is observed for the olefinic protons with a proton‐fluorine coupling constant ^3^ *J*_HF_=6.5 Hz, which is increased compared to that of the neutral compound ^3^ *J*_HF_=4.5 Hz. This indicates that, owing to the protonation, the electron withdrawing property of fluorine is reduced and the resonance‐electron‐donating ability is simultaneously increased.^[28]^ This observation is discussed in the section below in further detail. There is high correspondance between the NMR spectra of **1**, **2** and **4**. In *a*HF solution no difference between the monoprotonated and diprotonated fumaryl fluoride is observable due to rapid proton exchange with the superacidic solvent.[^9^, ^11^]

### Quantum chemical calculations

**Figure 6:**
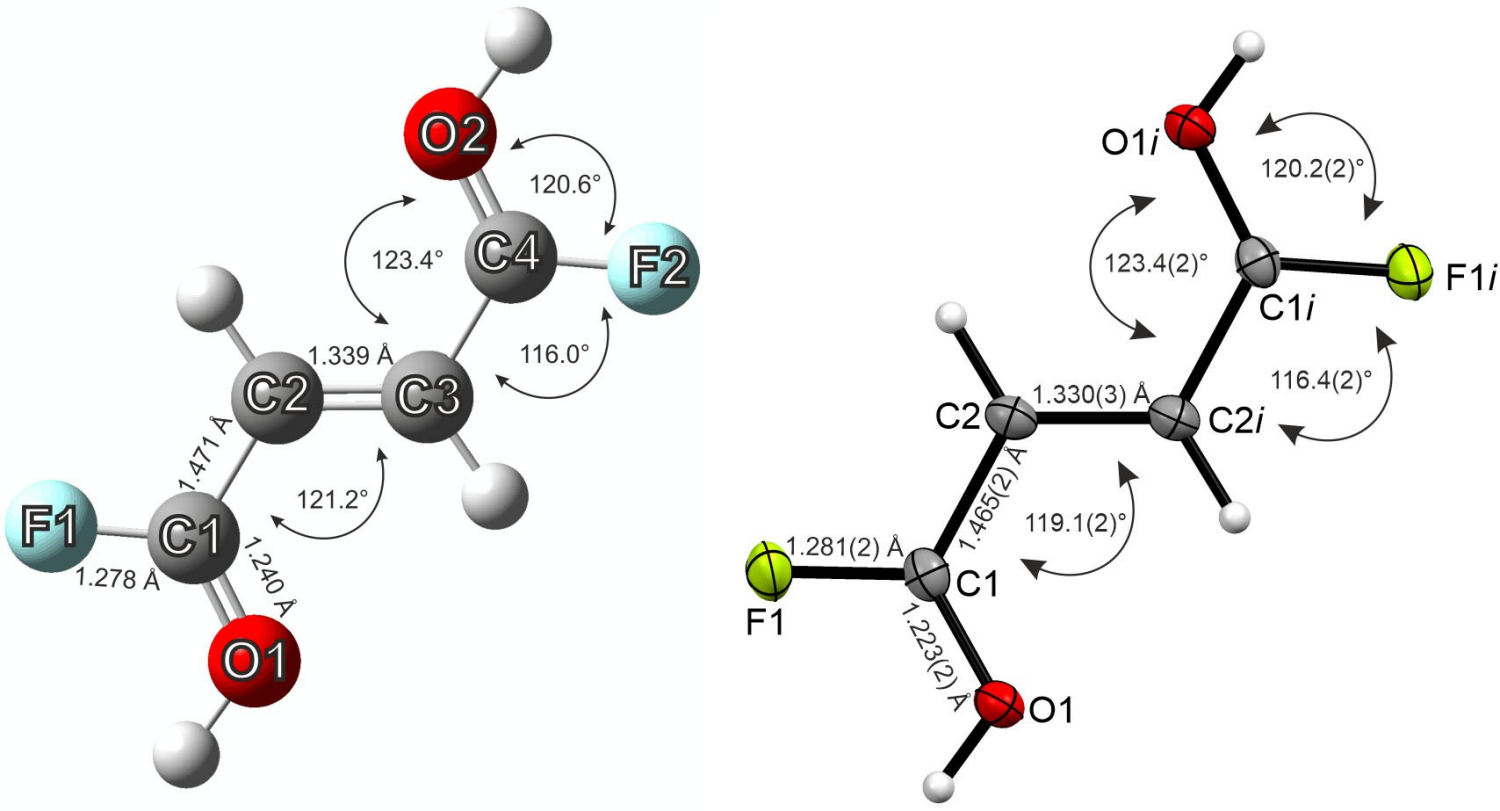
Calculated (left) and experimental (right) structures of [C_4_H_4_F_2_O_2_]^2+^ with bond lengths and angles. The calculation was performed for [C_4_H_4_F_2_O_2_ ⋅ 2 HF]^2+^, but the HF molecules are omitted for clarity.

All quantum chemical calculations were carried out at the B3LYP/aug‐cc‐pVTZ level of theory with the Gaussian program package. By comparing the calculated frequencies and structural parameters of the free cation [C_4_H_4_F_2_O_2_]^2+^ with the experimental data, large differences are shown (Tables S9 and S10). This is based on the hydrogen bonds confirmed by the crystal structure of **1** and **2**. To simulate the hydrogen bonds, HF molecules were added to the gas phase structure of the free cation, as it is established in the literature.^[23]^ The structural parameters of the HF complex of the cation [C_4_H_4_F_2_O_2_ ⋅ 2 HF]^2+^ agree well with the experimental data of **1** (Table S9) and the experimental vibrational frequencies of **1** are better described by the [C_4_H_4_F_2_O_2_ ⋅ 2 HF]^2+^ cation (Table S10). A comparison of the calculated structure of the [C_4_H_4_F_2_O_2_ ⋅ 2 HF]^2+^ cation with the experimental cationic structure of **1** is shown in Figure 6. The added HF molecules are omitted in the calculated structure for clarification. All experimentally obtained bond lengths and angles of the dication are in good agreement with the calculated structure. An exception is the C1−O1 bond distance, which is slightly overestimated in the calculation, compared to the experimental value. Presumably this is based on intermolecular interactions existing in the solid state, which are not taken into account comparably in the calculations.

**Figure 7:**
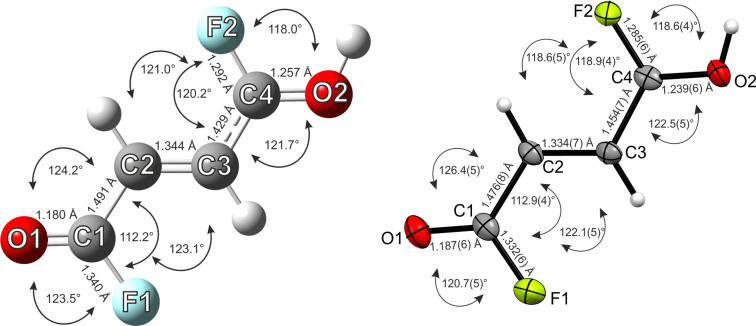
Calculated (left) and experimental (right) structures of [C_4_H_3_F_2_O_2_]^+^ with bond lengths and angles. The calculation was performed for [C_4_H_3_F_2_O_2_ ⋅ HF]^+^, but the HF molecule is omitted for clarity.

In the same fashion as for the dicationic species an HF complex^[23]^ was calculated for the monoprotonated cation [C_4_H_3_F_2_O_2_ ⋅ HF]^+^ to simulate the hydrogen bonds in the solid state. The calculated structural parameters agree well with the experimental data of **4** (Table S11, Supporting Information Information). In Figure 7, the calculated structure of [C_4_H_3_F_2_O_2_ ⋅ HF]^+^, in which the added HF molecule is omitted for clarification, and the monocation of the single‐crystal X‐ray structure are illustrated. Overall, the calculated and experimentally obtained parameters of the monocation are in good compliance. Though the C3−C4 bond distance is underestimated in the calculated structure of [C_4_H_3_F_2_O_2_ ⋅ HF]^+^, compared to the crystal structure of **4**.

**Figure 8:**
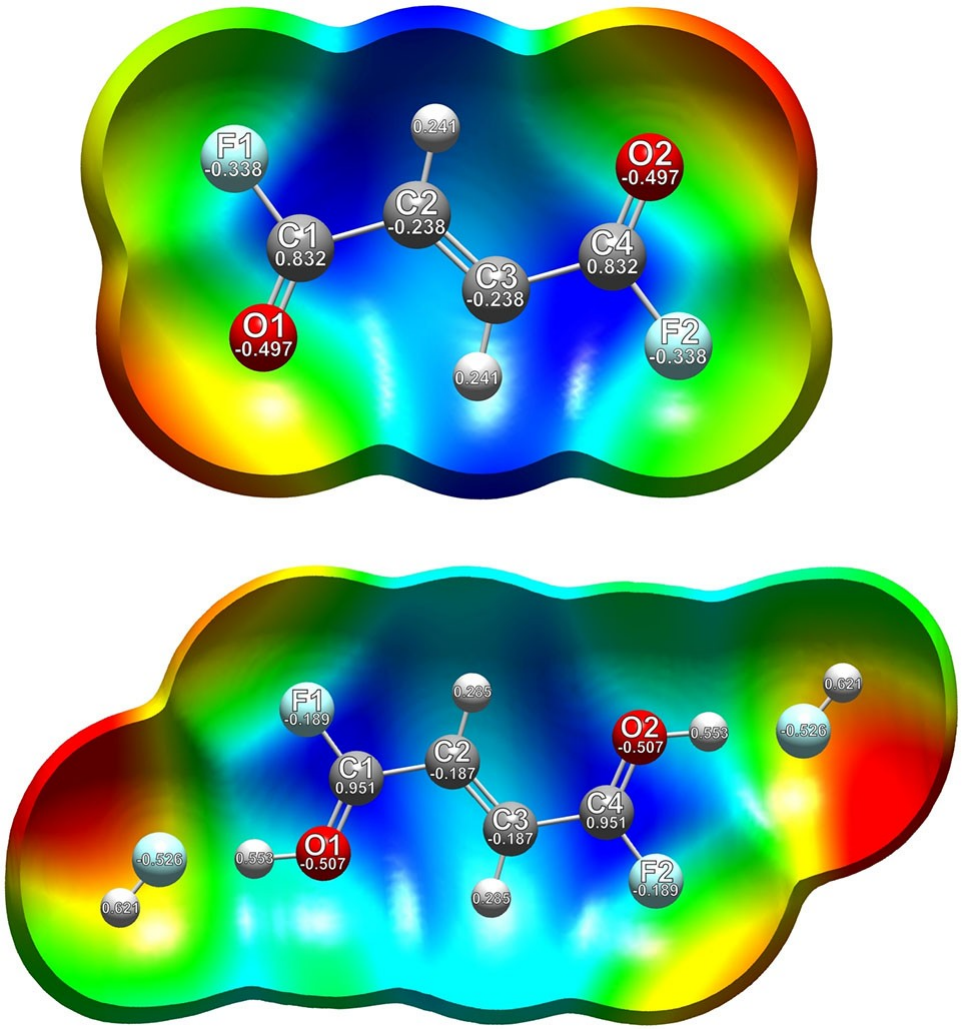
Top: Calculated ESP surface mapped onto an electron density isosurface value of 0.0004 bohr^−3^ with the color scale ranging from −0.029 a.u. (red) to 0.035 a.u. (blue) of *cis‐cis−C*_4_H_2_F_2_O_2_. Bottom: calculated ESP map with an electron density isosurface value of 0.00004 bohr^−3^ and a color scale range from −0.2 a.u. (red) to 0.275 a.u. (blue) of [C_4_H_4_F_2_O_2_ ⋅ 2 HF]^2+^. The NPA charges are given in [a.u.].

**Scheme 1:**
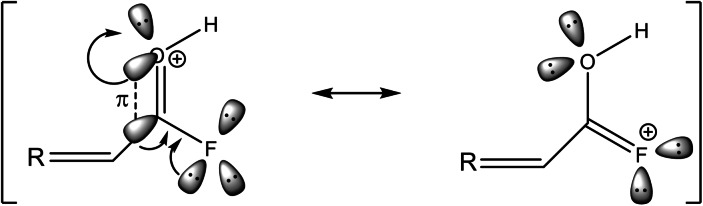
Illustration of the +R effect by using the example of a protonated acyl fluoride moiety.

As the protonation results in an elongation of the C=O bond lengths in contrast to fumaryl fluoride, the C−F bond distances are reduced, which is predicted by the quantum chemical calculations and confirmed by the vibrational spectra, as well as by the single‐crystal X‐ray structure analyses. This peculiarity of the protonated acyl fluoride moiety prompted us to further investigate the electron distribution and the charge‐related properties of the dication in comparison with the reactant. Thus, electrostatic potential (ESP) maps combined with natural population analysis (NPA) charges were calculated. In Figure 8, the ESP maps together with the NPA charges of *cis‐cis*‐fumaryl fluoride and [C_4_H_4_F_2_O_2_ ⋅ 2 HF]^2+^ are illustrated. The ESP map of the neutral compound shows a positive electrostatic potential along the carbon skeleton. The negative electrostatic potential is found on the oxygen and fluorine atoms, which also reveal the highest negative NPA charges. The C−F bond of the acyl fluoride group is polarized, increasing the positive charge density on the carbon atom, owing to the high electronegativity of fluorine.^[1]^ The electrostatic attraction between F^δ−^ and (O=C^δ+^) reduces the C−F bond distance,^[1]^ in comparison to regular C−F bond lengths (1.36 Å).^[14]^ The diprotonation of fumaryl fluoride results in a rearrangement of the electrostatic potential distribution. The positive electrostatic potential in the ESP map of [C_4_H_4_F_2_O_2_ ⋅ 2 HF]^2+^ is located on the carbonyl fluoride moiety. In comparison to the reactant, the positive NPA charges of the C1 and C4 atoms in the diprotonated species increase. This leads to the assumption, that the positive charges are delocalized over the carbonyl fluoride groups. By comparing the NPA charges of fumaryl fluoride with the NPA charges of the HF complex of the dication, the negative charges of the oxygen atoms increase slightly, whereas the negative charges of the fluorine atoms decrease considerably. The electron distribution shifts from the fluorine atom, which is inductively electron withdrawing but electron donating by resonance (+M effect) despite its high electronegativity,[^28^, ^29^, ^30^, ^31^, ^32^] to the C−F bond in the diprotonated cation. This resonance effect, that opposes the inductive effect, generating a more electropositive carbon, is established in literature as +R effect,[^33^, ^34^] which is illustrated in Scheme 1. The bond between carbon and fluorine reveals a low double bond character, which is described in the literature for several organofluorine compounds.[^28^, ^29^, ^30^, ^31^, ^32^, ^33^, ^34^] To draw comparisons of diprotonated fumaryl fluoride with organofluorine species, which are already related to resonance effects based on fluorine, the C−F bond lengths of CHF_3_ ^[35]^ and CF_3_Cl^[36]^ are consulted. The carbon fluorine bond distances in CHF_3_ (1.326(13) Å)^[35]^ and CF_3_Cl (1.328(2) Å)^[36]^ are considerably shortened in comparison to regular C−F bond lengths (1.36 Å).^[14]^ This strengthening of the C−F bond is explained by a decreasing ionic character of the bond.^[34]^ The great resonance‐electron‐donating ability of fluorine leads to C=F double bond structures gaining increased importance.[^28^, ^34^] Notably, the C−F bond distance in **1** is even shorter than those of CHF_3_ ^[35]^ and CF_3_Cl,^[36]^ and therefore the contributing structure with the C=F double bond is of higher importance. Finally, we assume that the strongest bond in organic chemistry^[1]^ is further strengthened due to the protonation of the acyl fluoride moiety, based on the resonance stabilization of the carbon–fluorine bond. This is especially reinforced for analogous diprotonated superelectrophiles.^[37]^

## Conclusions

Fumaryl fluoride has been examined in the superacidic systems XF/MF_5_ (X=H, D; M=As, Sb) for the first time. The salts of the monoprotonated and diprotonated fumaryl fluoride represent the first examples of a protonated acyl fluoride moiety. They were isolated and characterized by low‐temperature vibrational spectroscopy. Single‐crystal X‐ray structure analyses and low‐temperature NMR spectroscopy were performed for [C_4_H_3_F_2_O_2_]^+^[SbF_6_]^−^ and for [C_4_H_4_F_2_O_2_]^2+^([MF_6_]^−^)_2_ (M=As, Sb). The experimental results have been discussed together with quantum chemical calculations on the cations [C_4_H_4_F_2_O_2_ ⋅ 2 HF]^2+^ and [C_4_H_3_F_2_O_2_ ⋅ HF]^+^ at the B3LYP/aug‐cc‐pVTZ level of theory. The vibrational spectra and single‐crystal X‐ray structure analyses showed that the protonation results in an elongation of the C=O bonds, whereas the C−F bond lengths are considerably reduced. In order to investigate the electron distribution and the charge‐related properties of the diprotonated species, electrostatic potential (ESP) maps combined with natural population analysis (NPA) charges were calculated. The electron distribution shifts from the fluorine atom, which is inductively electron withdrawing, although it is electron donating by resonance,[^28^, ^29^, ^30^, ^31^, ^32^] to the C−F bond in the diprotonated cation, known in literature as the +R effect.^[33]^ In the NMR spectra of diprotonated fumaryl fluoride, the enhanced resonance‐electron‐donating ability is observed in the increased proton–fluorine coupling constants. The bond between carbon and fluorine reveals a low double‐bond character in the diprotonated cation. We conclude that the strongest bond in organic chemistry^[1]^ is further strengthened by the protonation of the acyl fluoride moiety on account of the +R effect.

## Experimental Section

***CAUTION***! Any contact with the components must be avoided. SbF_5_, AsF_5_ and the reported salts can produce HF upon contact with water, burning the skin and causing irreparable injury. Adequate precautionary measures must be taken when handling these materials.

**Syntheses of [C_4_H_4_F_2_O_2_]^2+^([AsF_6_]^−^)_2_ (1), [C_4_H_3_F_2_O_2_]^+^[AsF_6_]^−^ (3) and [C_4_H_2_D_2_F_2_O_2_]^2+^([AsF_6_]^−^)_2_ (5)**: At first, fumaryl fluoride (100 mg, 0.83 mmol) was condensed into a FEP reactor vessel at −196 °C. Additionally, arsenic pentafluoride (595 mg, 3.5 mmol **1**, **5**; 280 mg, 1.65 mmol **3**) and approximately 2 mL anhydrous hydrogen fluoride (*a*HF; **1**, **3**) deuterium fluoride (*a*DF; **5**), respectively, were condensed into the FEP reactor vessel at −196 °C. The reaction mixture was warmed up to −30 °C and homogenzied until the solution was clear. In dynamic vacuum excess HF was removed at −78 °C. The compounds were obtained as colorless crystalline solids with decomposition temperatures of 15 °C for **1**, **5** and −10 °C for **3**. The rector was left in an ethanol bath at −50 °C for crystallization of **1**.

**Syntheses of [C_4_H_4_F_2_O_2_]^2+^([SbF_6_]^−^)_2_ (2), [C_4_H_3_F_2_O_2_]^+^[SbF_6_]^−^ (4) and [C_4_H_2_D_2_F_2_O_2_]^2+^([SbF_6_]^−^)_2_ (6)**: Antimony pentafluoride (310 mg, 1.43 mmol) was condensed into a FEP reactor vessel at −196 °C. In addition, fumaryl fluoride (40 mg, 0.33 mmol **2**, **6**; 156 mg, 1.3 mmol **4**) and 2 mL of *a*HF (**2**, **4**) or *a*DF (**6**), respectively, were condensed into the reactor vessel at −196 °C. The reaction mixture was warmed up to −30 °C and homogenized until the formed salt was dissolved thoroughly. Excess HF was removed in dynamic vacuum at −78 °C. The compounds were obtained as colorless crystalline solids with decomposition temperatures of 15 °C for **2**, **6** and −10 °C for **4**. The rector was left in an ethanol bath at −50 °C for crystallization of **2** and at −40 °C for crystallization of **4**.

Deposition Numbers 2065273 (for **1**), 2065274 (for **2**) and 2065275 (for**4**) contain the supplementary crystallographic data for this paper. These data are provided free of charge by the joint Cambridge Crystallographic Data Centre and Fachinformationszentrum Karlsruhe Access Structures service.

## Conflict of interest

The authors declare no conflict of interests.

1

## Supporting information

As a service to our authors and readers, this journal provides supporting information supplied by the authors. Such materials are peer reviewed and may be re‐organized for online delivery, but are not copy‐edited or typeset. Technical support issues arising from supporting information (other than missing files) should be addressed to the authors.

Supporting Information

## Data Availability

The data that support the findings of this study are available in the supplementary material of this article.
