# Supplementary material for: Strengthening of the C−F Bond in Fumaryl Fluoride with Superacids
Source: Chemistry. 2022 Feb 10;28(15):e202104422. doi: 10.1002/chem.202104422 (PMC9303274; doi:10.1002/chem.202104422)
Supplement: Supplementary file 1 — Supporting Information [file CHEM-28-0-s001.pdf]

Chemistry–A European Journal

Supporting Information

Strengthening of the C–F Bond in Fumaryl Fluoride with Superacids

Marie C. Bayer, Christoph Kremser, Christoph Jessen, Alexander Nitzer, and Andreas J. Kornath*

Figure S1: Formula unit of $[C_4H_4F_2O_2]^{2+}([AsF_6]^-)_2$ (displacement ellipsoids with 50% probability). Symmetry operations: *i = 1–x, y, 0.5–z.*

Table S1: Selected bond lengths and angles of $[C_4H_4F_2O_2]^{2+}([AsF_6]^-)_2$ **(1)**. The estimated standard deviation is marked in parentheses. Symmetry operations: *i = 1–x, y, 0.5–z; iii = 1–x, 1–y, 1–z; iv = 1–x, –y, 1–z.*

Figure S2: Projection of the interatomic contacts in $[C_4H_4F_2O_2]^{2+}([AsF_6]^-)_2$ **(1)** (displacement ellipsoids 50% probability). Symmetry operations: *i = 1–x, y, 0.5–z; ii = x, 1–y, –0.5+z; iii = 1–x, 1–y, 1–z; iv = 1–x, –y, 1–z; v = x, –y, –0.5+z.* Interatomic contacts are drawn as dashed lines.

Figure S3: Detail of the crystal structure of $[C_4H_4F_2O_2]^{2+}([AsF_6]^-)_2$ **(1)** (displacement ellipsoids with 50% probability). Interatomic contacts are drawn as dashed lines.

Figure S4: Formula unit of $[C_4H_4F_2O_2]^{2+}([SbF_6]^-)_2$ (displacement ellipsoids with 50% probability). Symmetry operations: *i = 2–x, –y, 1–z.*

Table S2: Selected bond lengths and angles of $[C_4H_4F_2O_2]^{2+}([SbF_6]^-)_2$ **(2)**. The estimated standard deviation is marked in parentheses. Symmetry operations: *i = 2–x, –y, 1–z; ii = 2–x, 1–y, 1–z; iii = 1+x, –1+y, z.*

Figure S5: Projection of the interatomic contacts in $[C_4H_4F_2O_2]^{2+}([SbF_6]^-)_2$ **(2)** (displacement ellipsoids 50% probability). Symmetry operations: *i = 2–x, –y, 1–z; ii = 2–x, 1–y, 1–z; iii = 1+x, –1+y, z; iv = x, –1+y, z; v = 1–x, 1–y, 1–z*. Interatomic contacts are drawn as dashed lines.

Figure S6: Asymmetric unit of $[C_4H_3F_2O_2]^+[SbF_6]^-$ (displacement ellipsoids with 50% probability).

Table S3: Selected bond lengths and angles of $[C_4H_3F_2O_2]^+[SbF_6]^-$ **(4)**. The estimated standard deviation is marked in parentheses. Symmetry operations: *i = 0.5+x, 1.5–y, –0.5+z; iii = –1+x, 1–y, 0.5+z.*

Figure S7: Projection of the interatomic contacts in $[C_4H_3F_2O_2]^+[SbF_6]^-$ **(4)** (displacement ellipsoids 50% probability). Symmetry operations: *i = 0.5+x, 1.5–y, –0.5+z; ii = –0.5+x, 1.5–y, 0.5+z; iii = –1+x, 1–y, 0.5+z.* Interatomic contacts are drawn as dashed lines.

Figure S8: Detail of the crystal structure of $[C_4H_3F_2O_2]^+[SbF_6]^-$ **(4)** (displacement ellipsoids with 50% probability). Interatomic contacts are drawn as dashed lines.

Figure S9: Low-temperature IR and Raman spectra of $[C_4H_2D_2F_2O_2]^{2+}([AsF_6]^-)_2$ **(5)**, $[C_4H_2D_2F_2O_2]^{2+}([SbF_6]^-)_2$ **(6)** and fumaryl fluoride.[1]

Table S4: Experimental vibrational frequencies [cm^{-1}] of $[C_4H_4F_2O_2]^{2+}([MF_6]^-)_2$ (M = As, Sb) and calculated vibrational frequencies [cm^{-1}] of $[C_4H_4F_2O_2 \cdot 2\,HF]^{2+}$.

Table S5: Experimental vibrational frequencies [cm^{-1}] of $[C_4H_2D_2F_2O_2]^{2+}([MF_6]^-)_2$ (M = As, Sb) and calculated vibrational frequencies [cm^{-1}] of $[C_4H_2D_2F_2O_2 \cdot 2\,HF]^{2+}$.

Table S6: Experimental vibrational frequencies [cm^{-1}] of $[C_4H_3F_2O_2]^+[MF_6]^-$ (M = As, Sb) and calculated vibrational frequencies [cm^{-1}] of $[C_4H_3F_2O_2 \cdot HF]^+$.

Table S7: Observed ^1H, ^{13}C and ^{19}F chemical shifts [ppm] and spin-spin coupling constants [Hz] of fumaryl fluoride in $CDCl_3$.

Table S8: Selected observed ^1H, ^{13}C and ^{19}F NMR chemical shifts [ppm] and spin-spin coupling constants [Hz] of $C_4H_2F_2O_2$ in $CDCl_3$ as well as $C_4H_2F_2O_2$, **1**, **2** and **4** in *a*HF (external solvent Acetone-D_6), respectively.

Figure S10: Stacked ^1H NMR spectra of $C_4H_2F_2O_2$ dissolved in $CDCl_3$ and $C_4H_2F_2O_2$, $[C_4H_4F_2O_2]^{2+}([AsF_6]^-)_2$ **(1)**, $[C_4H_4F_2O_2]^{2+}([SbF_6]^-)_2$ **(2)** and $[C_4H_3F_2O_2]^+[SbF_6]^-$ **(4)** in *a*HF.

Figure S11: ^1H NMR spectrum of $C_4H_2F_2O_2$ at 26 °C in $CDCl_3$.

Figure S12: Detail of the ^1H NMR spectrum of $C_4H_2F_2O_2$ at 26 °C in $CDCl_3$.

Figure S13: ^{19}F NMR spectrum of $C_4H_2F_2O_2$ at 26 °C in $CDCl_3$.

Figure S14: Detail of the ^{19}F NMR spectrum of $C_4H_2F_2O_2$ at 26 °C in $CDCl_3$.

Figure S15: ^{13}C NMR spectrum of $C_4H_2F_2O_2$ at 26 °C in $CDCl_3$.

Figure S16: ^1H NMR spectrum of $C_4H_2F_2O_2$ at –41 °C in *a*HF and Acetone-D_6 as external solvent.

Figure S17: ^{19}F NMR spectrum of $C_4H_2F_2O_2$ at –41 °C in *a*HF and Acetone-D_6 as external solvent.

Figure S18: ^{13}C NMR spectrum of $C_4H_2F_2O_2$ at –41 °C in *a*HF and Acetone-D_6 as external solvent.

Figure S19: ^{1}H NMR spectrum of $[C_4H_4F_2O_2]^{2+}([AsF_6]^-)_2$ **(1)**, at –40 °C in *a*HF and Acetone-D_6 as external solvent.

Figure S20: ^{19}F NMR spectrum of $[C_4H_4F_2O_2]^{2+}([AsF_6]^-)_2$ **(1)**, at –40 °C in *a*HF and Acetone-D_6 as external solvent.

Figure S21: ^{13}C NMR spectrum of $[C_4H_4F_2O_2]^{2+}([AsF_6]^-)_2$ **(1)**, at –40 °C in *a*HF and Acetone-D_6 as external solvent.

Figure S22: ^{1}H NMR spectrum of $[C_4H_4F_2O_2]^{2+}([SbF_6]^-)_2$ **(2)**, at –40 °C in *a*HF and Acetone-D_6 as external solvent.

Figure S23: ^{19}F NMR spectrum of $[C_4H_4F_2O_2]^{2+}([SbF_6]^-)_2$ **(2)**, at –40 °C in *a*HF and Acetone-D_6 as external solvent.

Figure S24: ^{13}C NMR spectrum of $[C_4H_4F_2O_2]^{2+}([SbF_6]^-)_2$ **(2)**, at –35 °C in *a*HF and Acetone-D_6 as external solvent.

Figure S25: ^{1}H NMR spectrum of $[C_4H_3F_2O_2]^{+}[SbF_6]^-$ **(4)**, at –40 °C in *a*HF and Acetone-D_6 as external solvent.

Figure S26: ^{19}F NMR spectrum of $[C_4H_3F_2O_2]^{+}[SbF_6]^-$ **(4)**, at –40 °C in *a*HF and Acetone-D_6 as external solvent.

Figure S27: ^{13}C NMR spectrum of $[C_4H_3F_2O_2]^{+}[SbF_6]^-$ **(4)**, at –40 °C in *a*HF and Acetone-D_6 as external solvent.

Table S9: Calculated bond lengths and angles of the free cation $[C_4H_4F_2O_2]^{2+}$ and the HF complex of the cation $[C_4H_4F_2O_2 \cdot 2\ HF]^{2+}$ in comparison with the experimental structural parameters of $[C_4H_4F_2O_2]^{2+}([AsF_6]^-)_2$ **(1)**. The estimated standard deviation is marked in parentheses. Symmetry operations: *i = 1–x, y, 0.5–z*.

Table S10: Selected experimental vibrational frequencies [cm^{-1}] of $[C_4H_4F_2O_2]^{2+}([AsF_6]^-)_2$ **(1)** and calculated vibrational frequencies [cm^{-1}] of $[C_4H_4F_2O_2]^{2+}$ and $[C_4H_4F_2O_2 \cdot 2\ HF]^{2+}$.

Table S11: Calculated bond lengths and angles of the free cation $[C_4H_3F_2O_2]^{+}$ and the HF complex of the cation $[C_4H_3F_2O_2 \cdot HF]^{+}$ in comparison with the experimental structural parameters of $[C_4H_3F_2O_2]^{+}[SbF_6]^-$ **(4)**. The estimated standard deviation is marked in parentheses.

Table S12: Crystal data and structure refinement of $[C_4H_4F_2O_2]^{2+}([AsF_6]^-)_2$ **(1)**, $[C_4H_4F_2O_2]^{2+}([SbF_6]^-)_2$ **(2)** and $[C_4H_3F_2O_2]^{+}[SbF_6]^-$ **(4)**.

Table S13: Cartesian coordinates of calculated minimum structures of $[C_4H_4F_2O_2 \cdot 2\ HF]^{2+}$ at the B3LYP/aug-cc-pVTZ level of theory.

Table S14: Cartesian coordinates of calculated minimum structures of $[C_4H_4F_2O_2]^{2+}$ at the B3LYP/aug-cc-pVTZ level of theory.

Table S15: Cartesian coordinates of calculated minimum structures of $[C_4H_2D_2F_2O_2 \cdot 2\ HF]^{2+}$ at the B3LYP/aug-cc-pVTZ level of theory.

Table S16: Cartesian coordinates of calculated minimum structures of $[C_4H_3F_2O_2 \cdot HF]^{+}$ at the B3LYP/aug-cc-pVTZ level of theory.

Table S17: Cartesian coordinates of calculated minimum structures of $[C_4H_3F_2O_2]^{+}$ at the B3LYP/aug-cc-pVTZ level of theory.

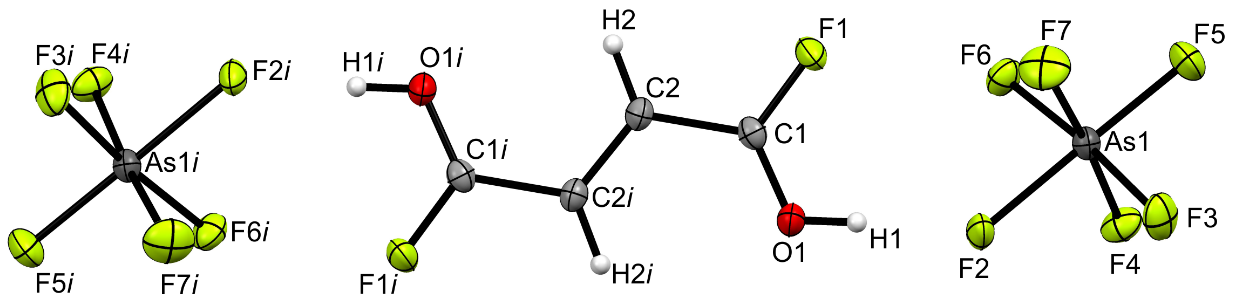

Figure S1: Formula unit of $[C_4H_4F_2O_2]^{2+}([AsF_6]^-)_2$ (displacement ellipsoids with 50% probability). Symmetry operations: *i = 1–x, y, 0.5–z.*

Table S1: Selected bond lengths and angles of $[C_4H_4F_2O_2]^{2+}([AsF_6]^-)_2$ (1). The estimated standard deviation is marked in parentheses. Symmetry operations: *i = 1–x, y, 0.5–z; iii = 1–x, 1–y, 1–z; iv = 1–x, –y, 1–z.*

Bond length [Å]	
C2–C2*i* (C=C)	1.330(3)
C1–C2 (C–C)	1.465(2)
C1–O1	1.223(2)
C1–F1	1.281(2)
**Bond angle [°]**	
O1–C1–F1	120.2(2)
O1–C1–C2	123.4(2)
F1–C1–C2	116.4(2)
C1–C2–C2*i*	119.1(2)
**Angle of torsion [°]**	
O1–C1–C2–C2*i*	9.5(3)
F1–C1–C2–C2*i*	–169.7(2)
C1–C2–C2*i*–C1*i*	177.9(1)
**Interatomic contact [Å]**	
O1–H1···F2	2.429(2)
C1···F4*iii*	2.614(2)
C1···F3*iv*	2.868(2)

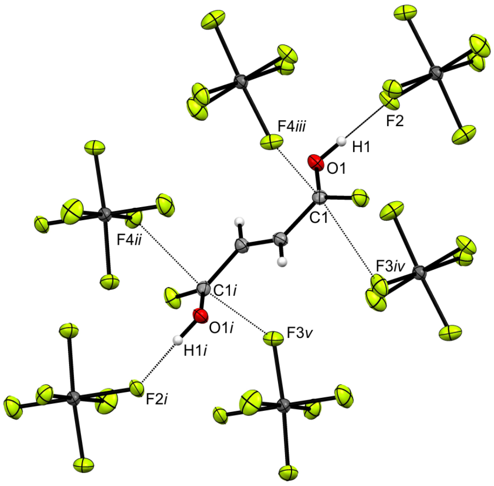

Figure S2: Projection of the interatomic contacts in $[C_4H_4F_2O_2]^{2+}([AsF_6]^-)_2$ (1) (displacement ellipsoids 50% probability). Symmetry operations: *i = 1–x, y, 0.5–z; ii = x, 1–y, –0.5+z; iii = 1–x, 1–y, 1–z; iv = 1–x, –y, 1–z; v = x, –y, –0.5+z.* Interatomic contacts are drawn as dashed lines.

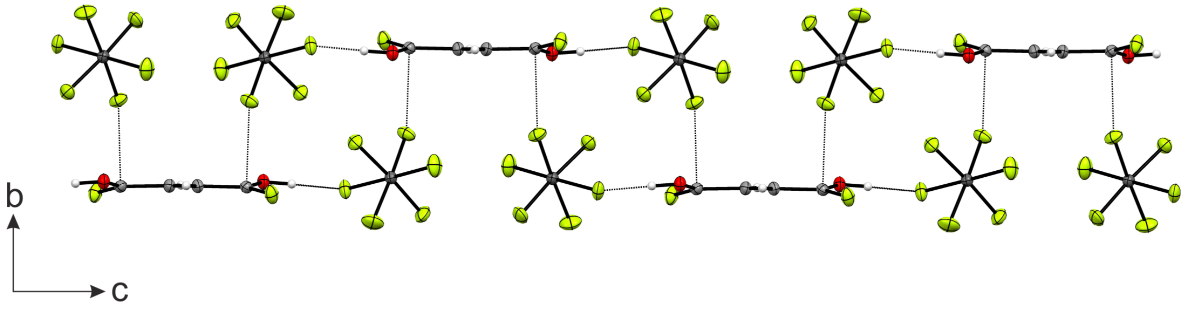

Figure S3: Detail of the crystal structure of $[C_4H_4F_2O_2]^{2+}([AsF_6]^-)_2$ (1) (displacement ellipsoids with 50% probability). Interatomic contacts are drawn as dashed lines.

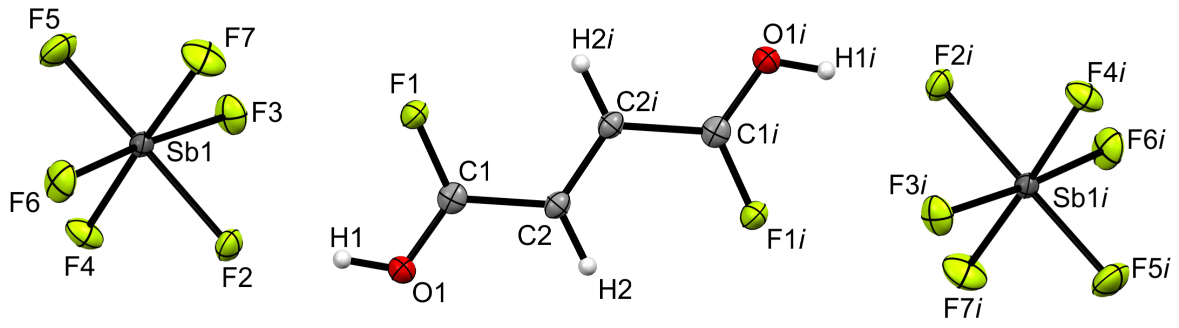

Figure S4: Formula unit of $[C_4H_4F_2O_2]^{2+}([SbF_6]^{-})_2$ (displacement ellipsoids with 50% probability). Symmetry operations: *i = 2–x, –y, 1–z.*

Table S2: Selected bond lengths and angles of $[C_4H_4F_2O_2]^{2+}([SbF_6]^{-})_2$ (2). The estimated standard deviation is marked in parentheses. Symmetry operations: *i = 2–x, –y, 1–z; ii = 2–x, 1–y, 1–z; iii = 1+x, –1+y, z.*

Bond length [Å]	
C2–C2*i* (C=C)	1.336(5)
C1–C2 (C–C)	1.461(4)
C1–O1	1.223(4)
C1–F1	1.287(4)
**Bond angle [°]**	
O1–C1–F1	120.6(3)
O1–C1–C2	121.1(3)
F1–C1–C2	118.2(3)
C1–C2–C2*i*	120.1(3)
**Angle of torsion [°]**	
O1–C1–C2–C2*i*	174.9(3)
F1–C1–C2–C2*i*	–6.0(5)
C1–C2–C2*i*–C1*i*	–180.0(3)
**Interatomic contact [Å]**	
O1–H1···F2	2.426(3)
C2–H2···F5*iii*	3.072(4)
C1···F3*ii*	2.578(4)

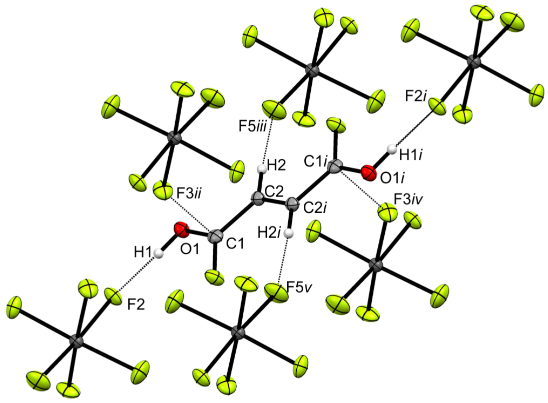

Figure S5: Projection of the interatomic contacts in $[C_4H_4F_2O_2]^{2+}([SbF_6]^-)_2$ (2) (displacement ellipsoids 50% probability). Symmetry operations: *i = 2–x, –y, 1–z; ii = 2–x, 1–y, 1–z; iii = 1+x, –1+y, z; iv = x, –1+y, z; v = 1–x, 1–y, 1–z*. Interatomic contacts are drawn as dashed lines.

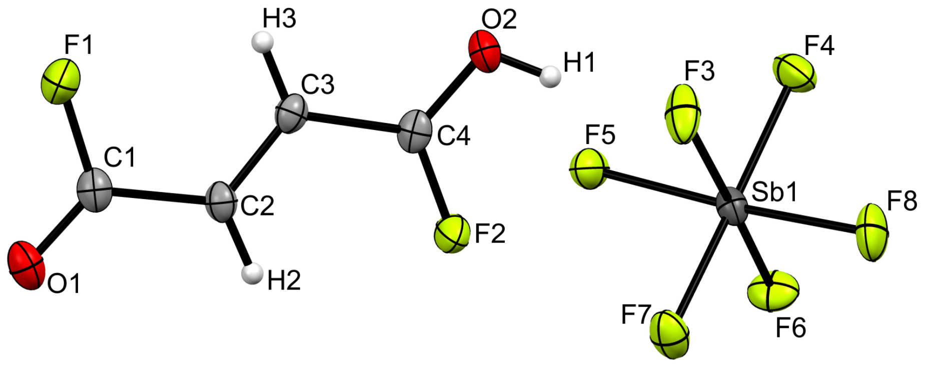

Figure S6: Asymmetric unit of $[C_4H_3F_2O_2]^{+}[SbF_6]^{-}$ (displacement ellipsoids with 50% probability).

Table S3: Selected bond lengths and angles of $[C_4H_3F_2O_2]^+[SbF_6]^-$ (4). The estimated standard deviation is marked in parentheses. Symmetry operations: *i = 0.5+x, 1.5–y, –0.5+z; iii = –1+x, 1–y, 0.5+z.*

Bond length [Å]	
C2–C3 (C=C)	1.334(7)
C1–C2 (C–C)	1.476(8)
C3–C4 (C–C)	1.454(7)
C1–O1 (C=O)	1.187(6)
C4–O2	1.239(6)
C1–F1	1.332(6)
C4–F2	1.285(6)
Bond angle [°]	
O1–C1–F1	120.7(5)
O2–C4–F2	118.6(4)
O1–C1–C2	126.4(5)
O2–C4–C3	122.5(5)
F1–C1–C2	112.9(4)
F2–C4–C3	118.9(4)
C1–C2–C3	122.1(5)
C4–C3–C2	118.6(5)
Angle of torsion [°]	
O1–C1–C2–C3	–174.5(5)
O2–C4–C3–C2	172.1(5)
F1–C1–C2–C3	7.1(7)
F2–C4–C3–C2	–6.2(7)
C1–C2–C3–C4	–177.1(5)
Interatomic contact [Å]	
O2–H1···F3	2.470(5)
C4···O1*i*	2.809(6)
C2···F8*iii*	2.839(7)

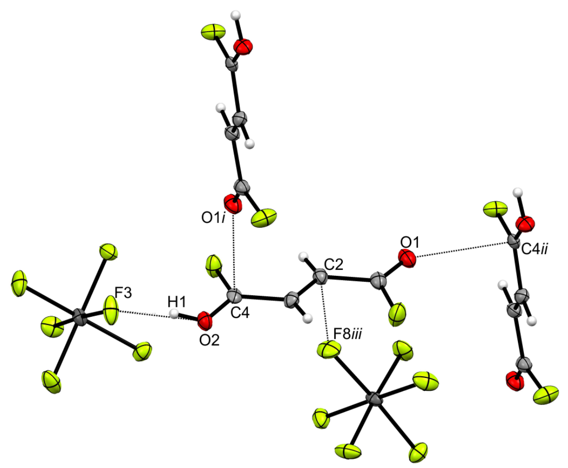

Figure S7: Projection of the interatomic contacts in $[C_4H_3F_2O_2]^+[SbF_6]^-$ (4) (displacement ellipsoids 50% probability). Symmetry operations: *i = 0.5+x, 1.5–y, –0.5+z; ii = –0.5+x, 1.5–y, 0.5+z; iii = –1+x, 1–y, 0.5+z.* Interatomic contacts are drawn as dashed lines.

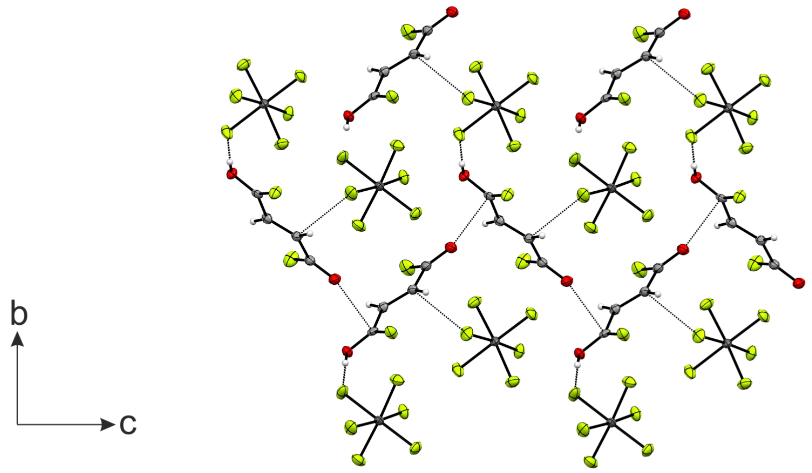

Figure S8: Detail of the crystal structure of $[C_4H_3F_2O_2]^+[SbF_6]^-$ (4) (displacement ellipsoids with 50% probability). Interatomic contacts are drawn as dashed lines.

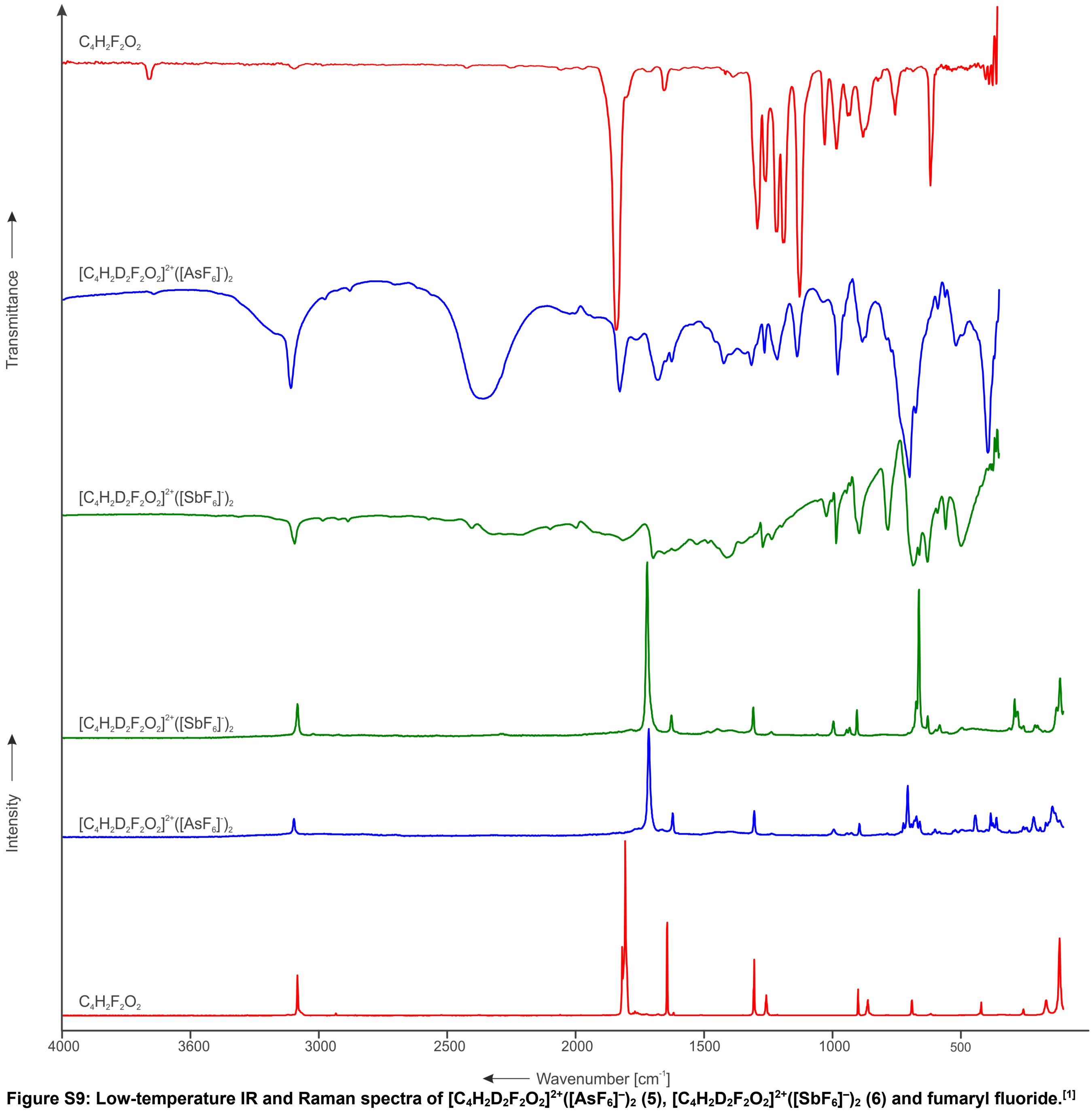

Figure S9: Low-temperature IR and Raman spectra of $[C_4H_2D_2F_2O_2]^{2+}([AsF_6]^-)_2$ (5), $[C_4H_2D_2F_2O_2]^{2+}([SbF_6]^-)_2$ (6) and fumaryl fluoride.[1]

Table S4: Experimental vibrational frequencies [cm^{-1}] of $[C_4H_4F_2O_2]^{2+}([MF_6]^-)_2$ (M = As, Sb) and calculated vibrational frequencies [cm^{-1}] of $[C_4H_4F_2O_2 \cdot 2\ HF]^{2+}$.

$[C_4H_4F_2O_2]^{2+}([AsF_6]^-)_2$ **(1)** exp.[a]		$[C_4H_4F_2O_2]^{2+}([SbF_6]^-)_2$ **(2)** exp.[a]		$[C_4H_4F_2O_2 \cdot 2\ HF]^{2+}$ calc.[b]	Assignment		
IR	Raman	IR	Raman	IR/Raman			
3109 vs		3094 vs		3190 (60/0)	ν_{21}	B_u	ν_{as}(C–H)
	3098 (24)		3085 (9)	3188 (0/79)	ν_1	A_g	ν_s(C–H)
3074 s							[c]
				2536 (3/246)	ν_2	A_g	ν_s(O–H)
3182 vs		3217 vs		2490 (7585/0)	ν_{22}	B_u	ν_{as}(O–H)
1880 w		1880 s					[c]
1823 m		1817 vs					[c]
	1717 (49)		1726 (21)	1713 (0/8)	ν_3	A_g	ν_s(C=O)
	1625 (42)		1629 (13)	1685 (0/384)	ν_4	A_g	ν(C=C)
1641 m		1666 vs		1681 (816/0)	ν_{23}	B_u	ν_{as}(C=O)
1450 m		1452 s		1485 (717/0)	ν_{24}	B_u	ν_{as}(C–F)
	1441 (8)		1445 (4)	1454 (0/4)	ν_5	A_g	ν_s(C–F)
1423 m		1423 s					[c]
1338 w		1365 s					[c]
	1304 (38)		1307 (19)	1337 (0/7)	ν_6	A_g	δ_s(CCH)
1317 w		1302 s		1302 (323/0)	ν_{25}	B_u	δ_{as}(CCH)
	1244 (20)	1246 s	1246 (10)	1280 (0/38)	ν_7	A_g	δ_s(COH)
1263 m		1273 s		1252 (216/0)	ν_{26}	B_u	δ_{as}(COH)
		1227 s					[c]
1217 m		1211 s					[c]
1153 w		1149 s		1023 (240/0)	ν_{12}	A_u	γ_{as}(COH)
		1022 vs		1022 (14/0)	ν_{17}	B_g	γ_s(COH)
999 w		982 vs		1020 (12/0)	ν_{13}	A_u	γ_{as}(HCCH)
974 m							[c]
	946 (24)		946 (18)	995 (0/6)	ν_8	A_g	ν_s(C–C)
	896 (23)		904 (15)	948 (0/4)	ν_{18}	B_g	γ_s(HCCH)
939 w							[c]
926 w		930 vs		933 (127/0)	ν_{27}	B_u	ν_{as}(C–C)
		887 s					[c]
		756 m		784 (12/0)	ν_{14}	A_u	γ_{as}(CCOF)
	693 (19)		692 (12)	712 (0/11)	ν_9	A_g	δ_s(COF)
	683 (11)		681 (19)				[c]
673 m		677 vs		647 (56/0)	ν_{28}	B_u	δ_{as}(COF)
	608 (8)		612 (4)	625 (0/1)	ν_{19}	B_g	γ_s(CCOF)
	589 (11)		588 (10)				[c]
554 vw		559 s		554 (46/0)	ν_{29}	B_u	δ_{as}(CCF)
	478 (7)		486 (5)	374 (0/2)	ν_{10}	A_g	δ_s(CCF)
	256 (16)		255 (6)	365 (0/2)	ν_{11}	A_g	δ_s(CCC)
	217 (33)		211 (6)				[c]
				190 (142/0)	ν_{15}	A_u	γ_{as}(CCC)
				136 (5/0)	ν_{30}	B_u	δ_{as}(CCC)
	133 (42)		129 (17)	132 (0/2)	ν_{20}	B_g	γ_s(CCC)
	115 (25)		113 (41)				[c]
				49 (15/0)	ν_{16}	A_u	τ(COF)
Vibrations of anions MF_6^- (M = As, Sb)							
	749 (9)						$[AsF_6]^-$
698 vs	708 (100)						$[AsF_6]^-$
	669 (47)	662 vs	662 (100)				$[AsF_6]^-$, $[SbF_6]^-$
625 m		629 vs	631 (12)				$[AsF_6]^-$, $[SbF_6]^-$
	594 (10)	592 m					$[AsF_6]^-$, $[SbF_6]^-$
501 w	500 (7)	498 s					$[AsF_6]^-$, $[SbF_6]^-$
390 s	384 (36)	440 m					$[AsF_6]^-$, $[SbF_6]^-$
368 w	374 (17)						$[AsF_6]^-$
	361 (29)	359 m	310 (5)				$[AsF_6]^-$, $[SbF_6]^-$
			290 (23)				$[SbF_6]^-$
			278 (15)				$[SbF_6]^-$
	247 (8)		266 (5)				$[AsF_6]^-$, $[SbF_6]^-$
			201 (7)				$[SbF_6]^-$
	145 (50)		172 (3)				$[AsF_6]^-$, $[SbF_6]^-$

[a] Abbrevations for IR intensities: v = very, s = strong, m = medium, w = weak. IR intensities in km/mol; Raman intensities in Å^4/u. Experimental Raman activities are relative to a scale of 1 to 100.
[b] Calculated on the B3LYP/aug-cc-pVTZ level of theory.
[c] Very probably combination tones/ overtones.

Table S5: Experimental vibrational frequencies [cm^{-1}] of $[C_4H_2D_2F_2O_2]^{2+}([MF_6]^-)_2$ (M = As, Sb) and calculated vibrational frequencies [cm^{-1}] of $[C_4H_2D_2F_2O_2 \cdot 2\,HF]^{2+}$.

$[C_4H_2D_2F_2O_2]^{2+}([AsF_6]^-)_2$ **(5)** exp.[a]		$[C_4H_2D_2F_2O_2]^{2+}([SbF_6]^-)_2$ **(6)** exp.[a]		$[C_4H_2D_2F_2O_2 \cdot 2\,HF]^{2+}$ calc.[b]	Assignment		
IR	Raman	IR	Raman	IR/Raman			
3109 m		3094 vs		3189 (60/0)	ν_{21}	B_u	ν_{as}(C–H)
	3098 (17)		3084 (19)	3187 (0/78)	ν_{1}	A_g	ν_{s}(C–H)
		2984 s					[c]
		2887 s					[c]
	2307 (2)	2403 s	2293 (2)	1869 (0/70)	ν_{2}	A_g	ν_{s}(O–D)
2359 s		2318 s		1830 (3909/0)	ν_{22}	B_u	ν_{as}(O–D)
		2098 s					[c]
		1996 s					[c]
1826 m		1815 vs					[c]
1763 w							[c]
	1715 (100)		1721 (100)	1712 (0/13)	ν_{3}	A_g	ν_{s}(C=O)
1678 m		1697 vs		1678 (927/0)	ν_{23}	B_u	ν_{as}(C=O)
1647 m	1662 (7)	1653 vs					[c]
1626 m	1622 (22)	1610 vs	1627 (13)	1676 (0/409)	ν_{4}	A_g	ν(C=C)
		1527 vs					[c]
		1483 vs	1486 (4)				[c]
	1447 (5)		1448 (5)				[c]
1421 m		1412 vs		1425 (703/0)	ν_{24}	B_u	ν_{as}(C–F)
	1403 (5)		1403 (4)	1402 (0/24)	ν_{5}	A_g	ν_{s}(C–F)
1313 m		1354 vs					[c]
	1304 (24)		1307 (18)	1320 (0/28)	ν_{6}	A_g	δ_{s}(CCH)
1263 w		1269 vs		1270 (56/0)	ν_{25}	B_u	δ_{as}(CCH)
1213 m	1237 (3)	1234 vs	1237 (4)				[c]
1136 w		1196 s					[c]
		1057 m	1058 (2)	1038 (0/6)	ν_{7}	A_g	δ_{s}(COD)
1034 vw		1022 s		1032 (57/0)	ν_{26}	B_u	δ_{as}(COD)
978 m		1001 m		1021 (53/0)	ν_{12}	A_u	γ_{as}(HCCH)
	995 (7)		996 (10)	962 (0/7)	ν_{8}	A_g	ν_{s}(C–C)
953 vw		984 vs					[c]
	942 (4)	943 m	944 (5)	948 (0/4)	ν_{17}	B_g	γ_{s}(HCCH)
	927 (4)	930 m	932 (6)				[c]
881 w	895 (12)	895 s	904 (16)	894 (223/0)	ν_{27}	B_u	ν_{as}(C–C)
870 w							[c]
	815 (3)						[c]
787 w	787 (4)	781 s		807 (84/0)	ν_{13}	A_u	γ_{as}(CCOF)
				759 (0/0)	ν_{18}	B_g	γ_{s}(COD)
770 w				720 (32/0)	ν_{14}	A_u	γ_{as}(COD)
	673 (19)		674 (21)	684 (0/9)	ν_{9}	A_g	δ_{s}(COF)
588 vw		590 s		629 (79/0)	ν_{28}	B_u	δ_{as}(COF)
	626 (4)		629 (12)	609 (0/1)	ν_{19}	B_g	γ_{s}(CCOF)
559 vw		557 s		552 (47/0)	ν_{29}	B_u	δ_{as}(CCF)
	582 (5)		582 (7)				[c]
	559 (4)		558 (3)				[c]
	496 (7)		495 (6)				[c]
393 vs	399 (7)	392 w		370 (0/2)	ν_{10}	A_g	δ_{s}(CCF)
366 w		361 vw					[c]
	309 (6)		310 (6)	360 (0/2)	ν_{11}	A_g	δ_{s}(CCC)
	256 (10)		255 (7)				[c]
	191 (9)		192 (5)	188 (150/0)	ν_{15}	A_u	γ_{as}(CCC)
	169 (13)		172 (4)	135 (5/0)	ν_{30}	B_u	δ_{as}(CCC)
	132 (23)		126 (17)	132 (0/2)	ν_{20}	B_g	γ_{s}(CCC)
	115 (16)		114 (34)	48 (15/0)	ν_{16}	A_u	τ(COF)
Vibrations of anions MF_6^- (M = As, Sb)							
	734 (5)						$[AsF_6]^-$
	723 (13)						$[AsF_6]^-$
	707 (48)						$[AsF_6]^-$
698 vs	691 (12)						$[AsF_6]^-$
673 s	681 (16)	685 vs					$[AsF_6]^-$, $[SbF_6]^-$
	659 (15)	660 vs	662 (84)				$[AsF_6]^-$, $[SbF_6]^-$
	600 (7)	629 vs	598 (5)				$[AsF_6]^-$, $[SbF_6]^-$
	529 (6)						$[AsF_6]^-$
517 w	522 (7)						$[AsF_6]^-$
	465 (7)	498 vs	456 (5)				$[AsF_6]^-$, $[SbF_6]^-$
	442 (20)						$[AsF_6]^-$
	421 (6)						$[AsF_6]^-$
	383 (22)	380 w					$[AsF_6]^-$, $[SbF_6]^-$
	375 (13)	372 w					$[AsF_6]^-$, $[SbF_6]^-$
	361 (18)		290 (22)				$[AsF_6]^-$, $[SbF_6]^-$
	248 (9)		279 (15)				$[AsF_6]^-$, $[SbF_6]^-$
	243 (9)		268 (6)				$[AsF_6]^-$, $[SbF_6]^-$
	216 (19)		211 (7)				$[AsF_6]^-$, $[SbF_6]^-$
	143 (28)		200 (7)				$[AsF_6]^-$, $[SbF_6]^-$

[a] Abbrevations for IR intensities: v = very, s = strong, m = medium, w = weak. IR intensities in km/mol; Raman intensities in Å^4/u. Experimental Raman activities are relative to a scale of 1 to 100.
[b] Calculated on the B3LYP/aug-cc-pVTZ level of theory.
[c] Very probably combination tones/ overtones.

Table S6: Experimental vibrational frequencies [cm^{-1}] of $[C_4H_3F_2O_2]^+[MF_6]^-$ (M = As, Sb) and calculated vibrational frequencies [cm^{-1}] of $[C_4H_3F_2O_2 \cdot HF]^+$.

$[C_4H_3F_2O_2]^+[AsF_6]^-$ **(3)** exp.[a]		$[C_4H_3F_2O_2]^+[SbF_6]^-$ **(4)** exp.[a]		$[C_4H_3F_2O_2 \cdot HF]^+$ calc.[b]	Assignment		
IR	Raman	IR	Raman	IR/Raman			
		3337 vs					[c]
		3109 vs		3212 (26/29)	ν_1	A'	ν(C–H)[d]
	3090 (17)		3084 (11)	3199 (6/54)	ν_2	A'	ν(C–H)
3196 vs		3203 vs		3061 (2649/166)	ν_3	A'	ν(O–H)[d]
1803 m	1823 (37)	1813 s	1825 (97)	1872 (180/260)	ν_4	A'	ν(C=O)
	1637 (56)		1647 (54)	1675 (636/227)	ν_5	A'	ν(C=C)
1770 m	1761 (100)	1770 w	1757 (60)	1621 (181/2)	ν_6	A'	ν(C=O)[d]
			1739 (55)				[c]
	1378 (14)		1354 (17)	1489 (195/12)	ν_7	A'	ν(C–F)[d]
	1304 (41)		1305 (28)	1316 (7/29)	ν_8	A'	δ(CCH)
1290 m	1284 (10)	1292 w	1281 (13)	1280 (22/1)	ν_9	A'	δ(CCH)
1261 m	1265 (8)	1261 w	1252 (8)	1259 (205/24)	ν_{10}	A'	δ(COH)[d]
1205 m		1209 m	1227 (5)	1213 (433/9)	ν_{11}	A'	ν(C–F)
1188 m		1186 m					[c]
1121 w		1121 w					[c]
1040 m		1038 w		1026 (32/1)	ν_{20}	A''	γ(HCCH)
987 w	981 (7)	987 w	982 (4)	956 (27/12)	ν_{12}	A'	ν(C–C)[d]
	962 (5)		946 (6)	944 (0/3)	ν_{21}	A''	γ(HCCH)
922 m	935 (13)	920 m	936 (7)	916 (122/0)	ν_{22}	A''	γ(COH)[d]
885 w	899 (24)	887 w	898 (26)	882 (34/13)	ν_{13}	A'	ν(C–C)
874 w			879 (6)				[c]
			856 (5)				[c]
	764 (6)	756 w	763 (4)	775 (12/1)	ν_{23}	A''	γ(CCOF)
	712 (23)		715 (18)				[c]
	691 (43)		691 (11)	687 (6/8)	ν_{14}	A'	δ(COF)
619 s		621 s	620 (5)	618 (22/0)	ν_{15}	A'	δ(COF)
	588 (12)		587 (4)	614 (5/1)	ν_{24}	A''	γ(CCOF)
548 w		546 vw		553 (18/1)	ν_{16}	A'	δ(CCF)
	263 (11)		266 (10)	252 (6/2)	ν_{17}	A'	δ(CCF)
			216 (12)	222 (20/1)	ν_{18}	A'	δ(CCC)
			170 (18)	170 (8/1)	ν_{25}	A''	γ(CCC)
	135 (52)		144 (39)	149 (32/0)	ν_{26}	A''	γ(CCC)
			108 (57)	127 (6/0)	ν_{19}	A'	δ(CCC)
				38 (1/0)	ν_{27}	A''	τ(COF)
Vibrations of anions MF_6^- (M = As, Sb)							
698 vs	699 (32)						$[AsF_6]^-$
	682 (48)		681 (9)				$[AsF_6]^-$, $[SbF_6]^-$
677 vs		671 s	671 (17)				$[AsF_6]^-$, $[SbF_6]^-$
			662 (19)				$[SbF_6]^-$
			648 (100)				$[SbF_6]^-$
			575 (9)				$[SbF_6]^-$
			566 (7)				$[SbF_6]^-$
			418 (6)				$[SbF_6]^-$
390 s	393 (11)	397 w					$[AsF_6]^-$, $[SbF_6]^-$
		384 w					$[SbF_6]^-$
	370 (27)	372 w					$[AsF_6]^-$, $[SbF_6]^-$
359 vw		365 vw					$[AsF_6]^-$, $[SbF_6]^-$
	309 (8)		296 (16)				$[AsF_6]^-$, $[SbF_6]^-$
			284 (25)				$[SbF_6]^-$
			279 (23)				$[SbF_6]^-$

[a] Abbrevations for IR intensities: v = very, s = strong, m = medium, w = weak. IR intensities in km/mol; Raman intensities in Å^4/u. Experimental Raman activities are relative to a scale of 1 to 100.

[b] Calculated on the B3LYP/aug-cc-pVTZ level of theory.

[c] Very probably combination tones/ overtones.

[d] protonated acyl fluoride moiety.

Table S7: Observed ^{1}H, ^{13}C and ^{19}F chemical shifts [ppm] and spin-spin coupling constants [Hz] of fumaryl fluoride in $CDCl_3$.

NMR Nucleus	Substance	Solvent	Temperature	Spectrometer Frequency	Chemical Shift δ [ppm] (Multiplicity, Coupling Constant, Integral, Assignment)
^{1}H	$C_4H_2F_2O_2$	$CDCl_3$	26 °C	400 MHz	6.97 (ddd, $^3J_{HH}$ = 12.6 Hz, $^3J_{HF}$ = 4.5 Hz, $^4J_{HF}$ = 2.7 Hz, 2H)<br>^{13}C-Satellites:<br>**Pair 1:** 7.21 ppm (d, $^5J_{FF}$ = 7.3 Hz) and 6.77 ppm (d, $^5J_{FF}$ = 7.3 Hz)<br>**Pair 2:** 7.17 ppm (d, $^5J_{FF}$ = 7.2 Hz) and 6.73 ppm (d, $^5J_{FF}$ = 7.3 Hz)<br>$^1J(^{13}C,^1H)$ = 175.5 Hz<br>$^2J(^{13}C,^1H)$ = 33.2 Hz
^{13}C	$C_4H_2F_2O_2$	$CDCl_3$	26 °C	101 MHz	153.57 (dd, $^1J_{CF}$ = 345.7 Hz, $^4J_{CF}$ = 1.5 Hz)<br>133.45 (dd, $^2J_{CF}$ = 71.3 Hz, $^3J_{CF}$ = 4.3 Hz)
^{19}F	$C_4H_2F_2O_2$	$CDCl_3$	26 °C	377 MHz	31.42 (dd, $^3J_{HF}$ = 4.5 Hz, $^4J_{HF}$ = 2.6 Hz)<br>^{13}C-Satellites:<br>**Pair 1:** 32.24 ppm (d, $^5J_{FF}$ = 7.4 Hz) and 30.32 ppm (d, $^5J_{FF}$ = 8.1 Hz)<br>**Pair 2:** 31.76 ppm (dd, $^3J_{HF}$ = 4.6 Hz, $^4J_{HF}$ = 2.9 Hz) and 30.84 ppm (dd, $^3J_{HF}$ = 4.5 Hz, $^4J_{HF}$ = 2.7 Hz)<br>$^2J(^{13}C,^1H)$ = 33.2 Hz

Table S8: Selected observed ^{1}H, ^{13}C and ^{19}F NMR chemical shifts [ppm] and spin-spin coupling constants [Hz] of $C_4H_2F_2O_2$ in $CDCl_3$ as well as $C_4H_2F_2O_2$, 1, 2 and 4 in *a*HF (external solvent Acetone-D_6), respectively.

	$C_4H_2F_2O_2$ (in $CDCl_3$)	$C_4H_2F_2O_2$ (in *a*HF, external solvent Acetone-D_6)	**1** (in *a*HF, external solvent Acetone-D_6)	**2** (in *a*HF, external solvent Acetone-D_6)	**4** (in *a*HF, external solvent Acetone-D_6)
^{1}H Chemical Shift δ [ppm] (Multiplicity, Coupling Constant, Assignment)	6.97 (ddd, $^{3}J_{HH}$ = 12.6 Hz, $^{3}J_{HF}$ = 4.5 Hz, $^{4}J_{HF}$ = 2.7 Hz, 2H)	6.70 (s, 2H)	9.99 (s, OH) 7.20 (dd, $^{3}J_{HF}$ = 6.4 Hz, $^{4}J_{HF}$ = 2.9 Hz, 2H)	10.05 (s, OH) 7.29 (dd, $^{3}J_{HF}$ = 6.5 Hz, $^{4}J_{HF}$ = 2.9 Hz, 2H)	9.99 (s, OH) 7.26 (dd, $^{3}J_{HF}$ = 6.5 Hz, $^{4}J_{HF}$ = 2.9 Hz, 2H)
^{13}C Chemical Shift δ [ppm] (Multiplicity, Coupling Constant, Assignment)	153.57 (dd, $^{1}J_{CF}$ = 345.7 Hz, $^{4}J_{CF}$ = 1.5 Hz, **C**OF) 133.45 (dd, $^{2}J_{CF}$ = 71.3 Hz, $^{3}J_{CF}$ = 4.3 Hz, **C**H)	157.81 (d, $^{1}J_{CF}$ = 349.0 Hz, **C**OF) 133.49 (dd, $^{2}J_{CF}$ = 64.1 Hz, $^{3}J_{CF}$ = 5.2 Hz, **C**H)	166.33 (d, $^{1}J_{CF}$ = 359.6 Hz, **C**(OH)F) 136.50 (dd, $^{2}J_{CF}$ = 49.1 Hz, $^{3}J_{CF}$ = 7.5 Hz, **C**H)	166.66 (d, $^{1}J_{CF}$ = 359.7 Hz, **C**(OH)F) 136.60 (dd, $^{2}J_{CF}$ = 48.7 Hz, $^{3}J_{CF}$ = 7.6 Hz, **C**H)	166.83 (d, $^{1}J_{CF}$ = 360.1 Hz, **C**(OH)F) 136.59 (dd, $^{2}J_{CF}$ = 48.4 Hz, $^{3}J_{CF}$ = 7.6 Hz, **C**H)
^{19}F Chemical Shift δ [ppm] (Multiplicity, Coupling Constant, Assignment)	31.42 (dd, $^{3}J_{HF}$ = 4.5 Hz, $^{4}J_{HF}$ = 2.6 Hz)	27.77 (s)	30.71 (s)	30.65 (s) –125.24 (s,br, $[SbF_6]^-$)	30.74 (s) –126.70 (s, br, $[SbF_6]^-$)

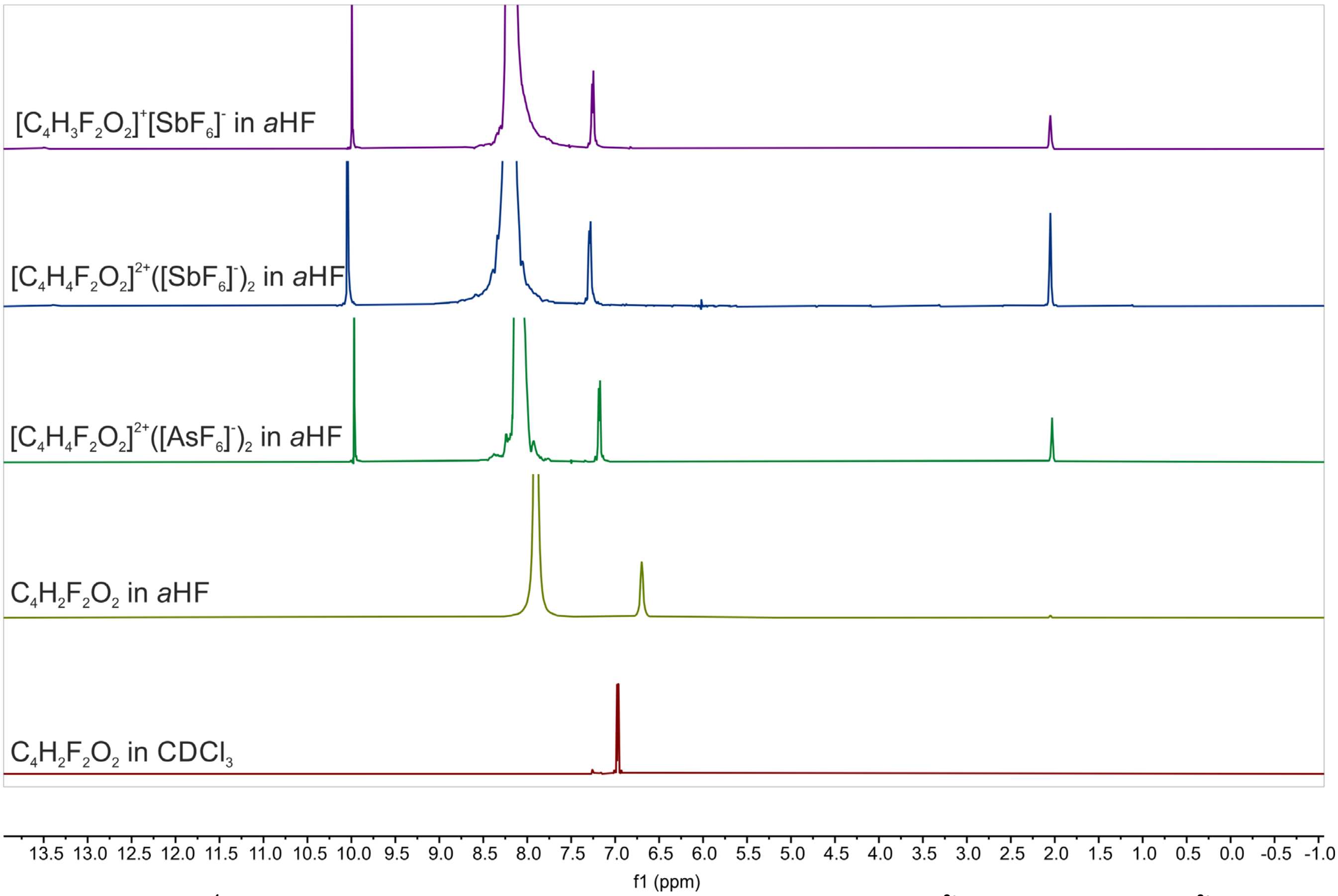

Figure S10: Stacked ^{1}H NMR spectra of $C_4H_2F_2O_2$ dissolved in $CDCl_3$ and $C_4H_2F_2O_2$, $[C_4H_4F_2O_2]^{2+}([AsF_6]^-)_2$ (1), $[C_4H_4F_2O_2]^{2+}([SbF_6]^-)_2$ (2) and $[C_4H_3F_2O_2]^+[SbF_6]^-$ (4) in *a*HF.

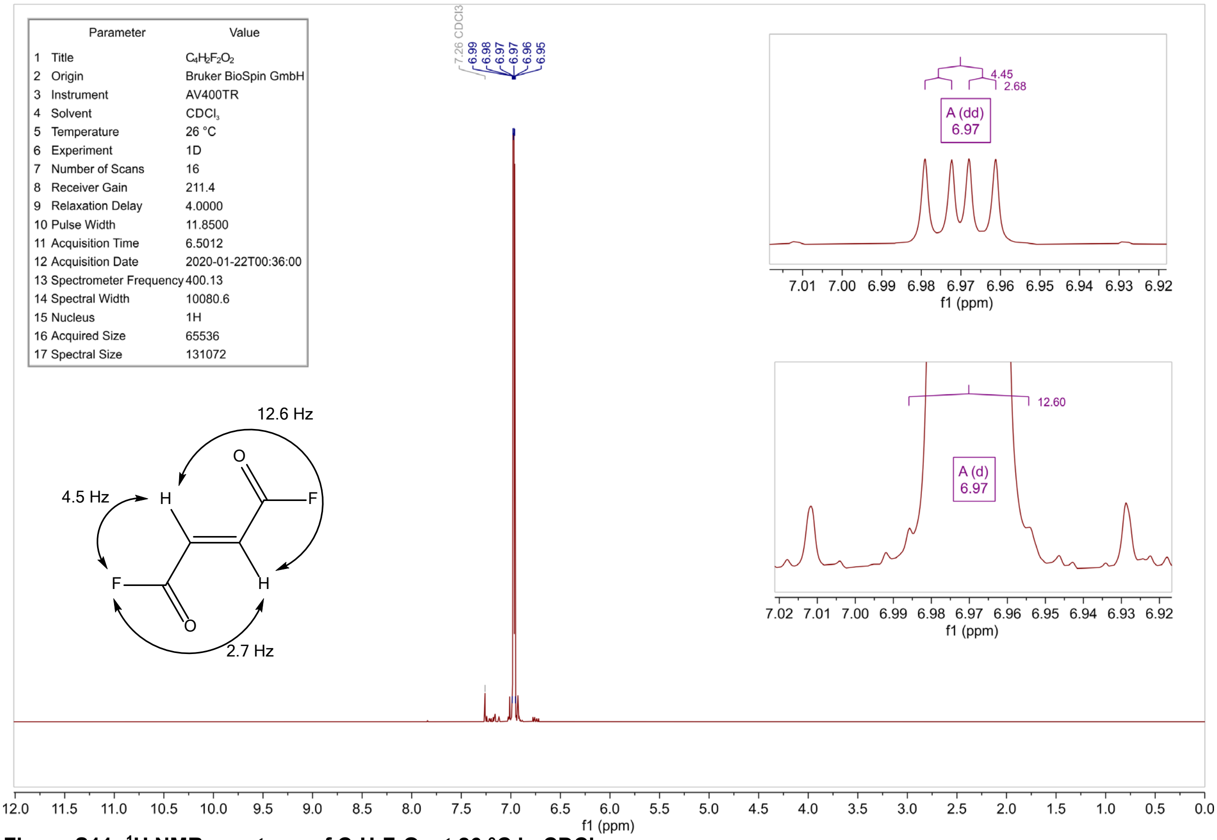

Figure S11: ^{1}H NMR spectrum of $C_4H_2F_2O_2$ at 26 °C in $CDCl_3$.

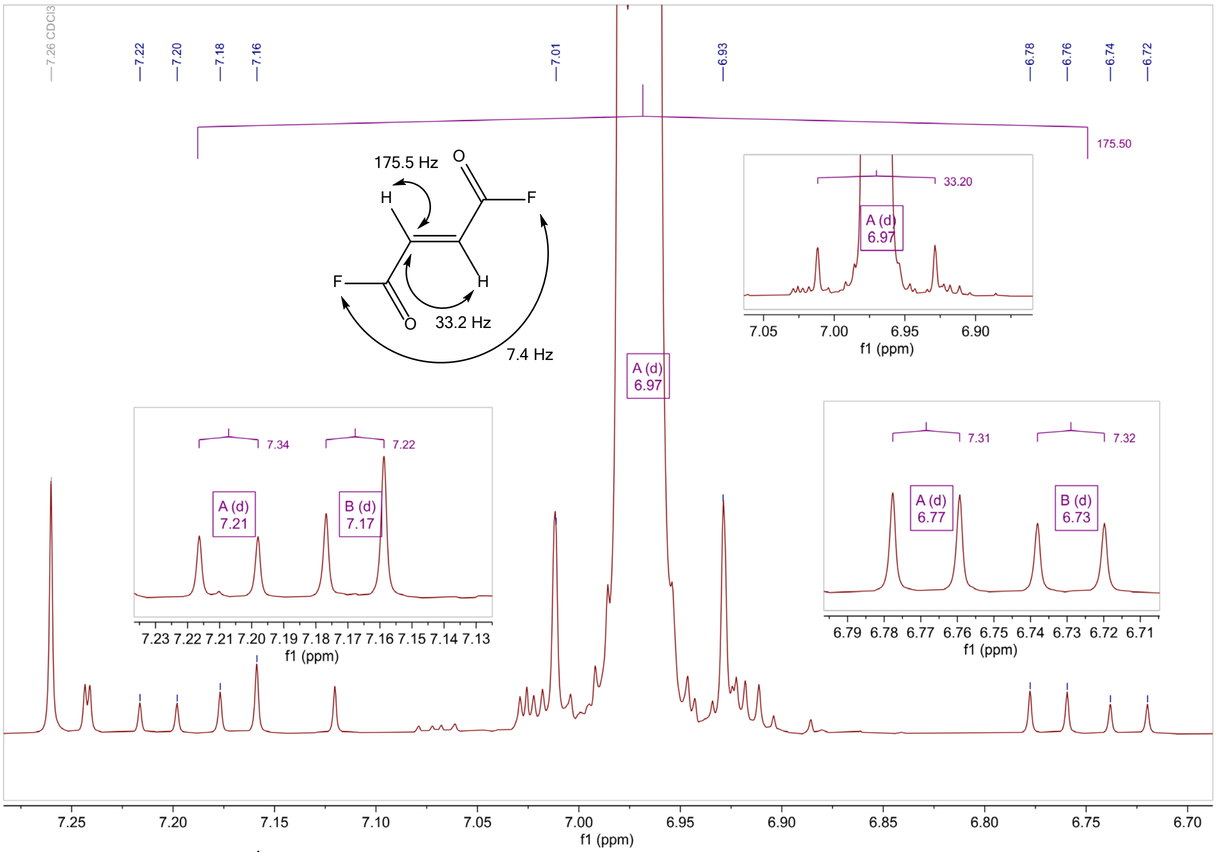

Figure S12: Detail of the ^{1}H NMR spectrum of $C_4H_2F_2O_2$ at 26 °C in $CDCl_3$.

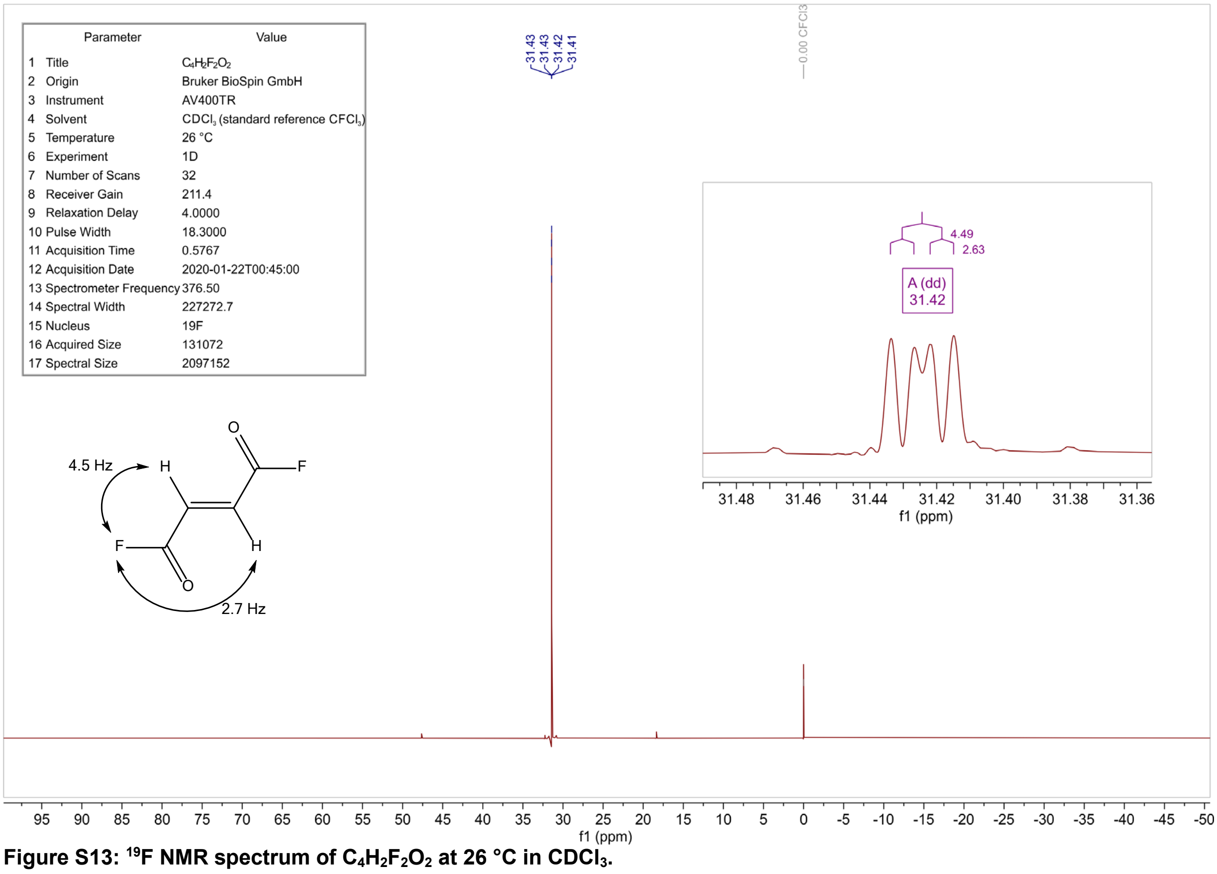

Figure S13: ^{19}F NMR spectrum of $C_4H_2F_2O_2$ at 26 °C in $CDCl_3$.

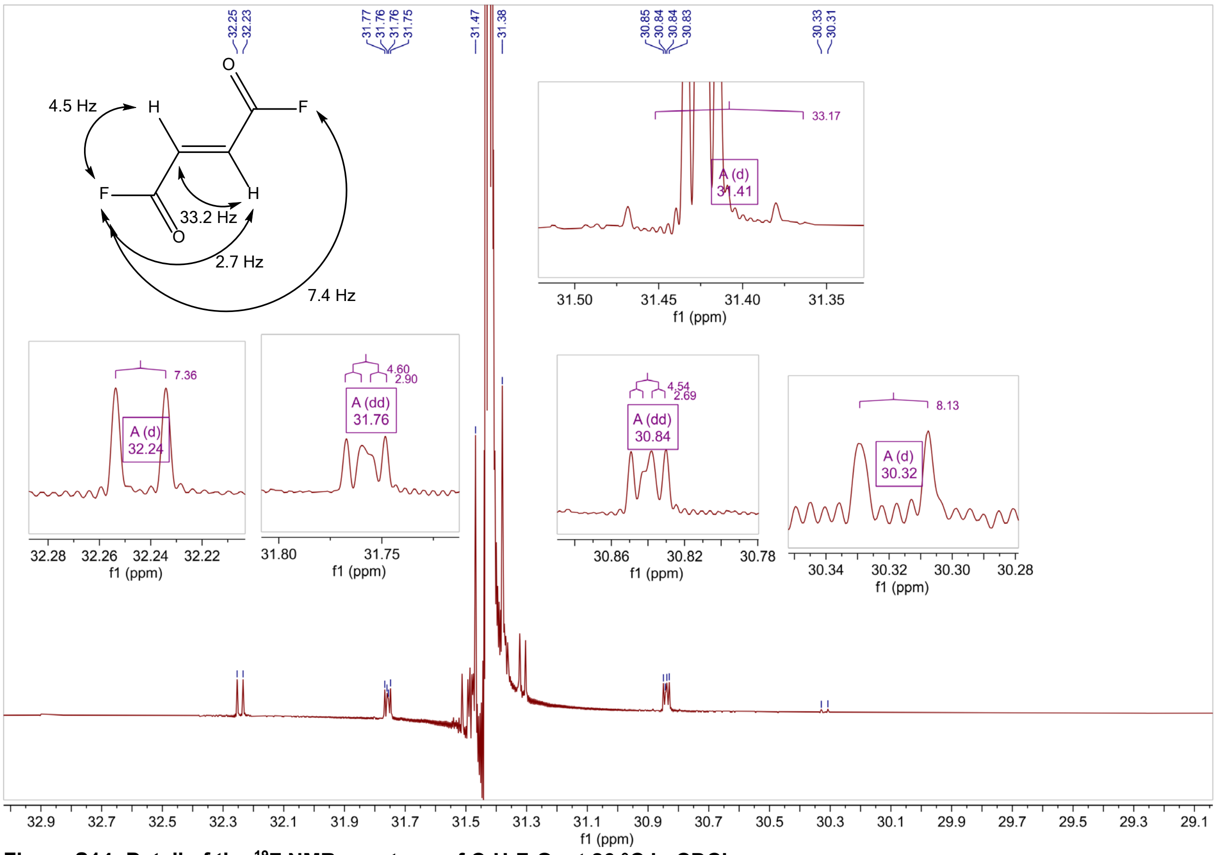

Figure S14: Detail of the ^{19}F NMR spectrum of $C_4H_2F_2O_2$ at 26 °C in $CDCl_3$.

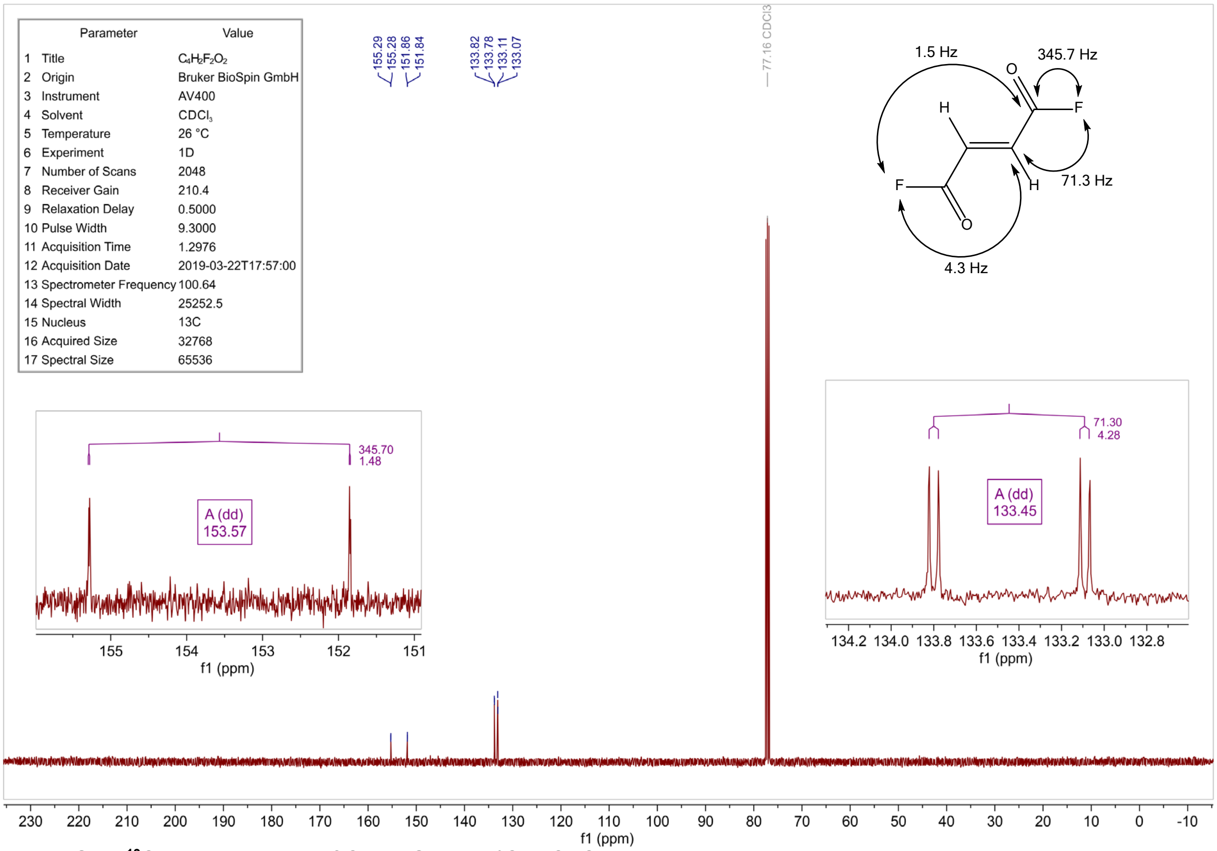

Figure S15: ^{13}C NMR spectrum of $C_4H_2F_2O_2$ at 26 °C in $CDCl_3$.

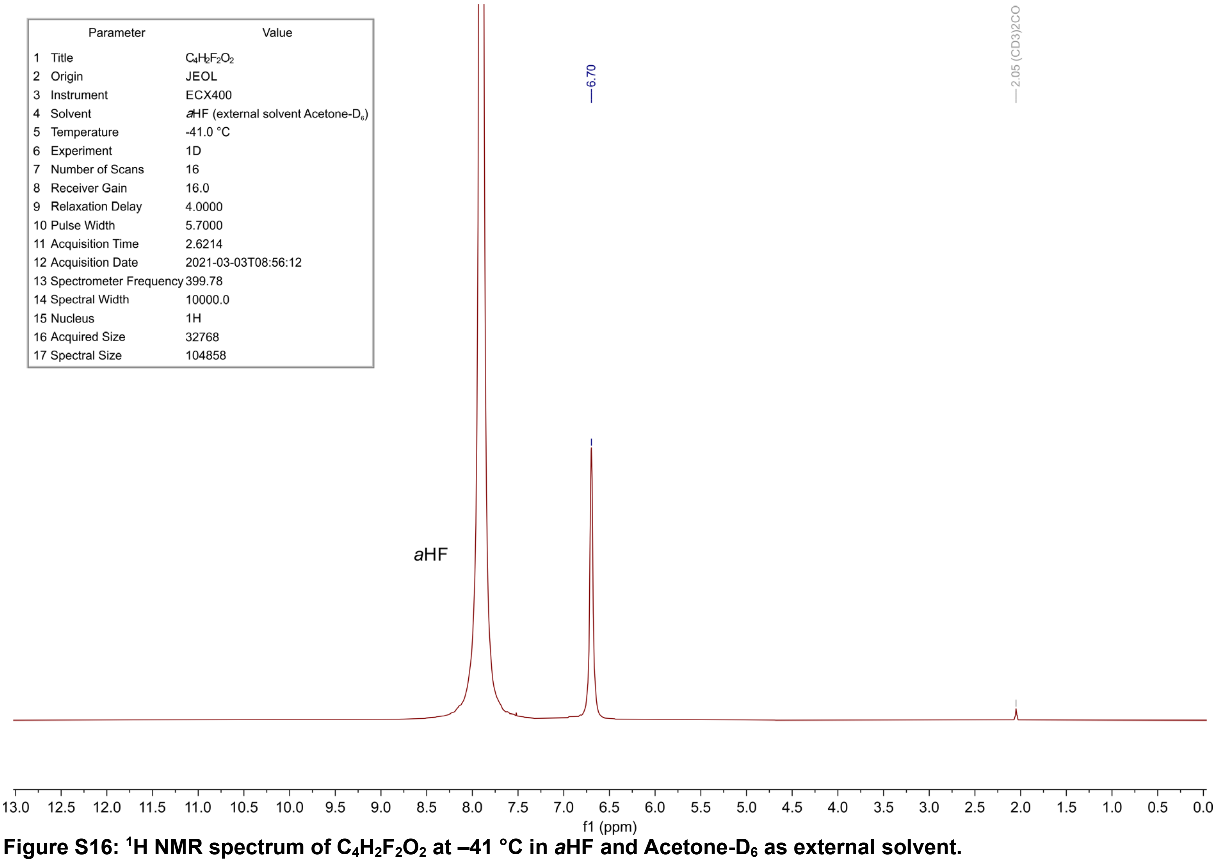

Figure S16: ^{1}H NMR spectrum of $C_4H_2F_2O_2$ at –41 °C in *a*HF and Acetone-D_6 as external solvent.

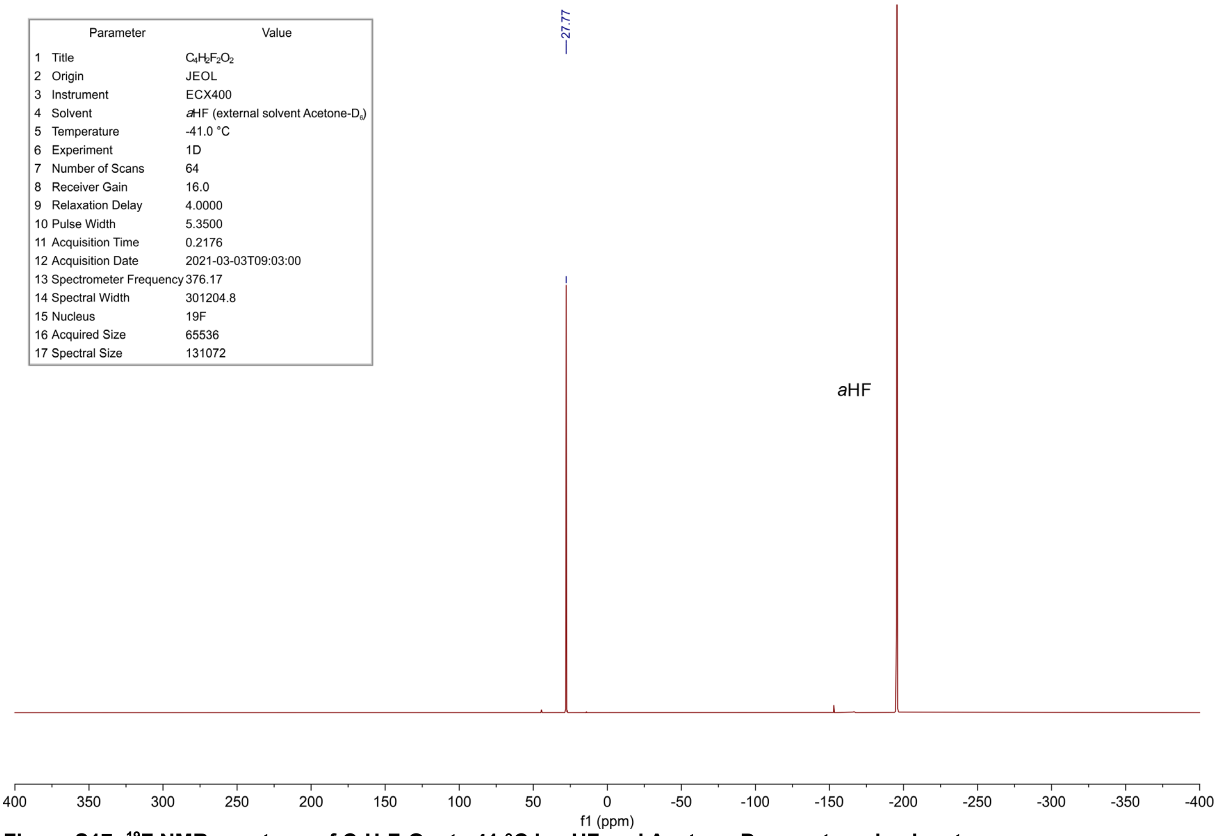

Figure S17: ^{19}F NMR spectrum of $C_4H_2F_2O_2$ at –41 °C in *a*HF and Acetone-D$_6$ as external solvent.

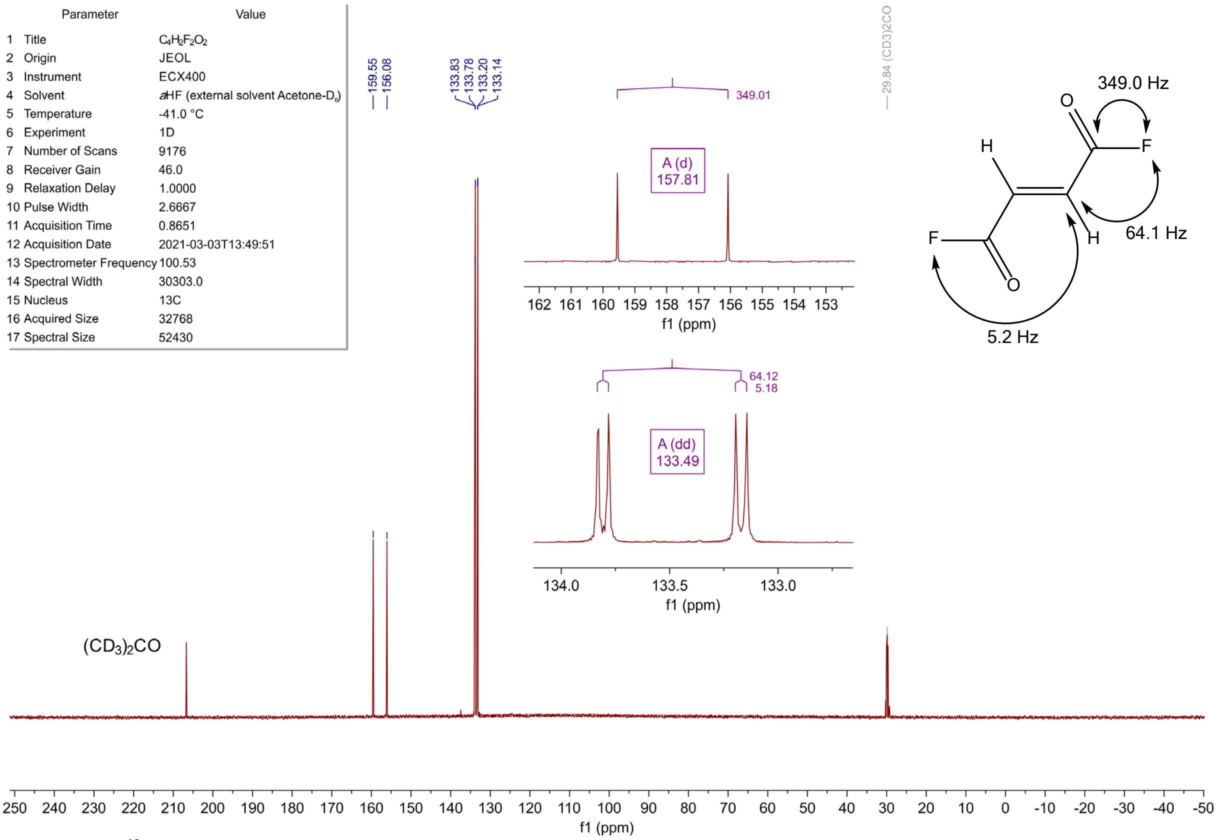

Figure S18: ^{13}C NMR spectrum of $C_4H_2F_2O_2$ at –41 °C in *a*HF and Acetone-D$_6$ as external solvent.

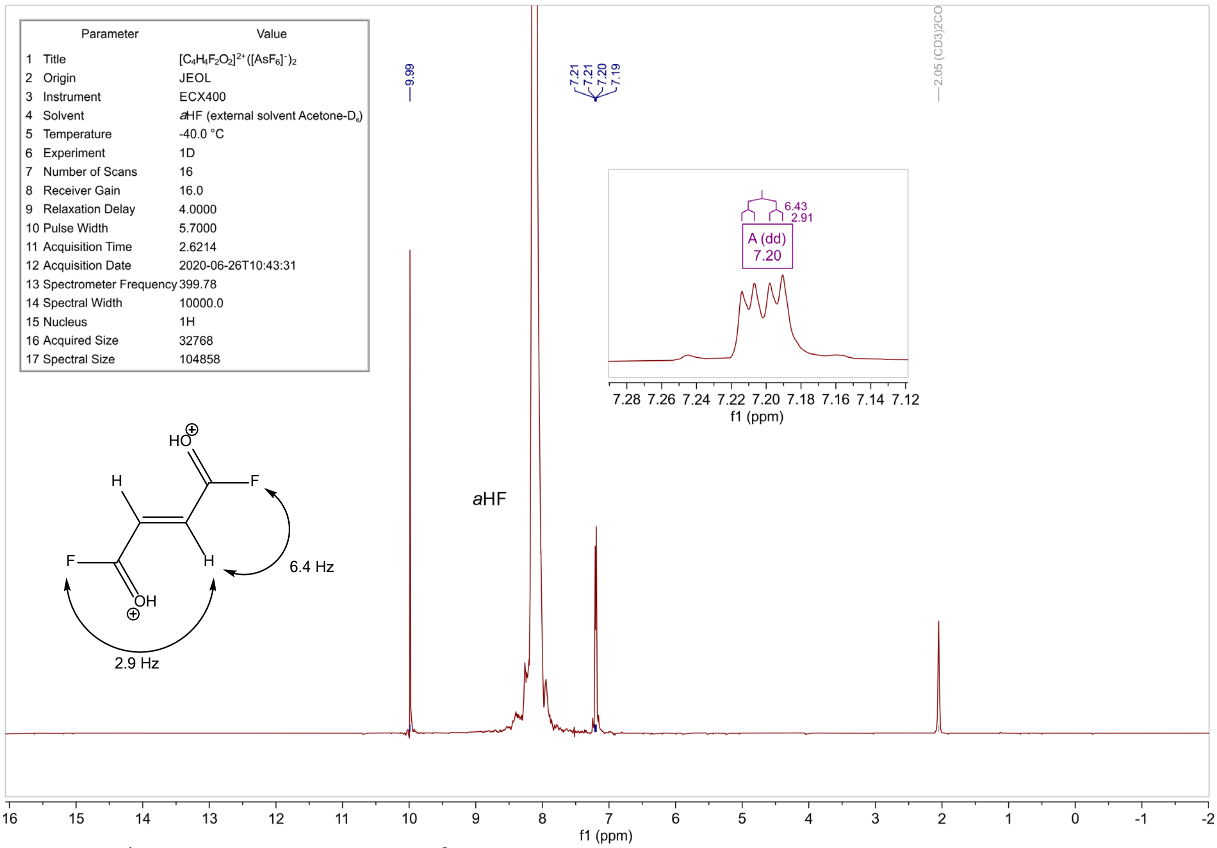

Figure S19: ^{1}H **NMR spectrum of** $[C_4H_4F_2O_2]^{2+}([AsF_6]^-)_2$ **(1), at –40 °C in** ***a*****HF and Acetone-D$_6$ as external solvent.**

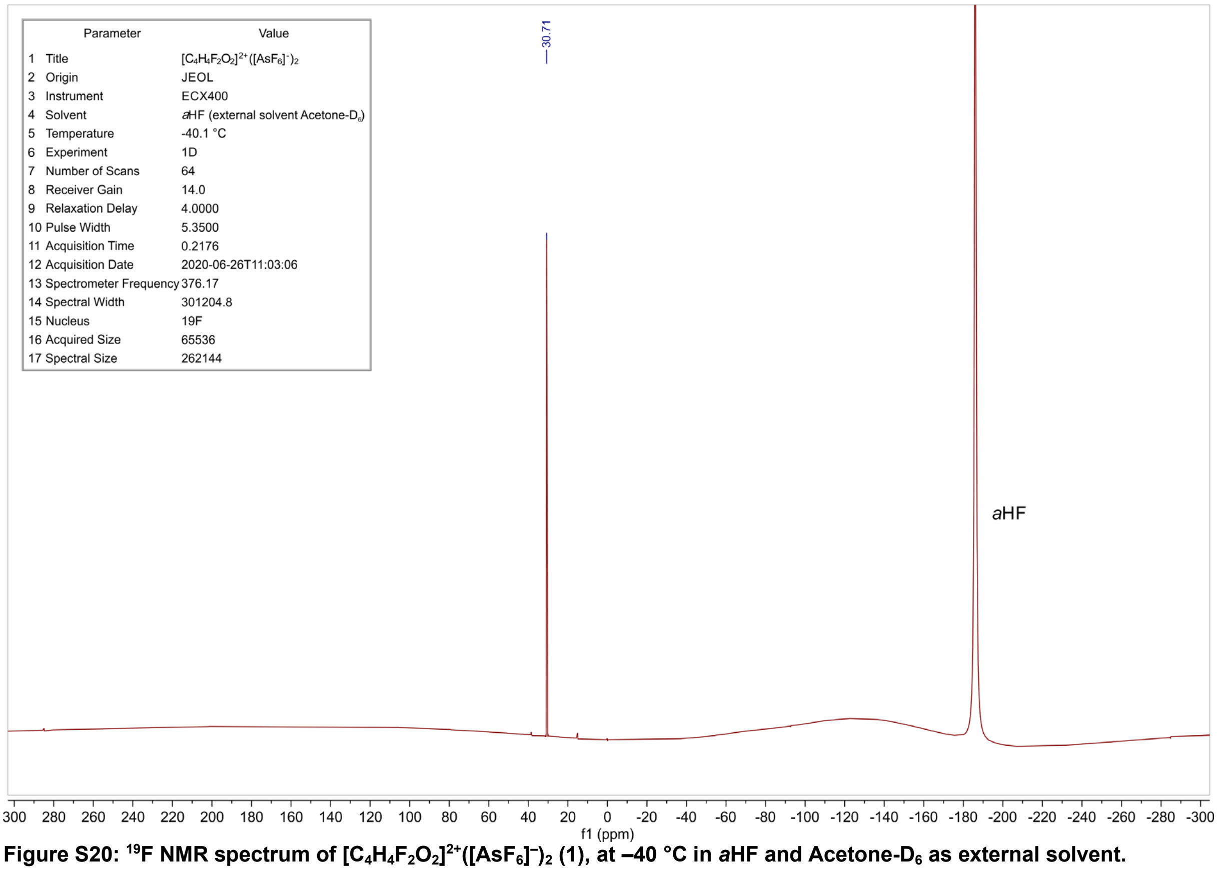

Figure S20: ^{19}F **NMR spectrum of** $[C_4H_4F_2O_2]^{2+}([AsF_6]^-)_2$ **(1), at –40 °C in** ***a*****HF and Acetone-D$_6$ as external solvent.**

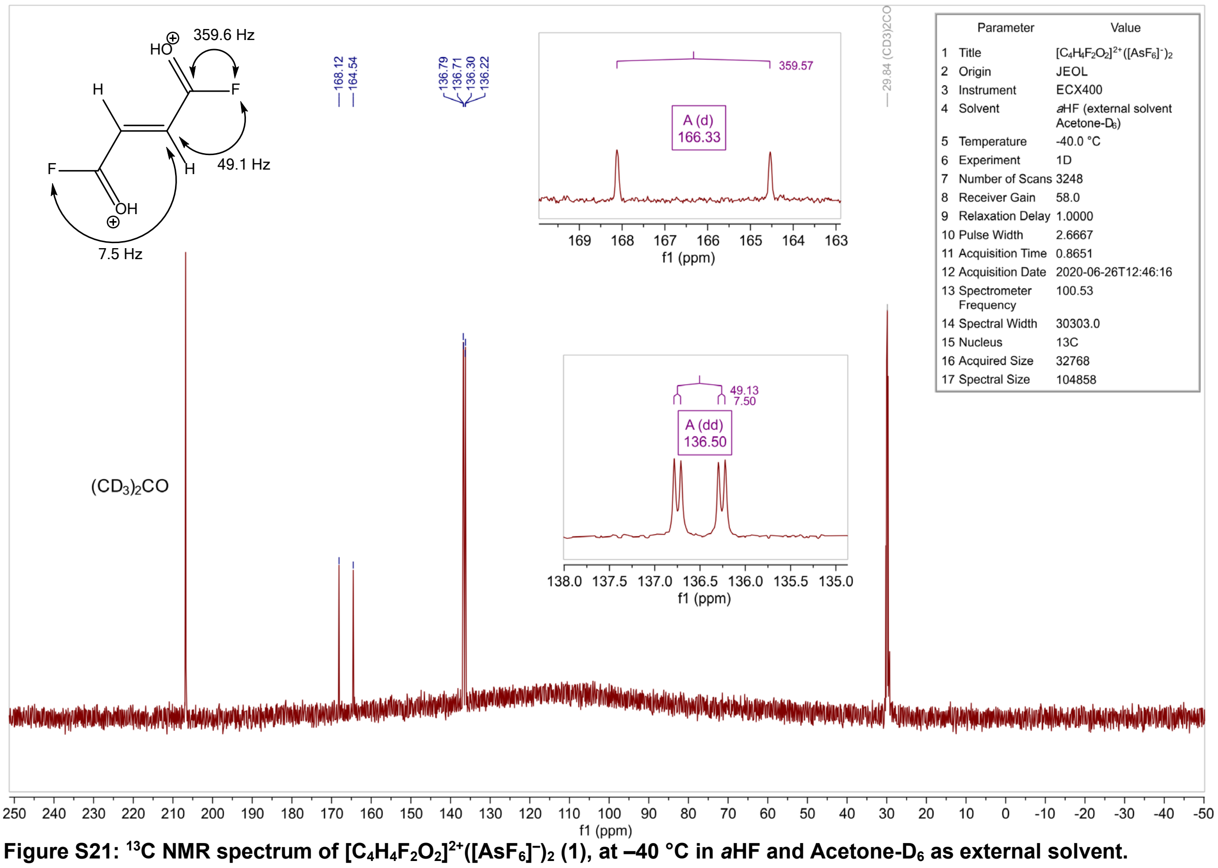

Figure S21: ^{13}C NMR spectrum of $[C_4H_4F_2O_2]^{2+}([AsF_6]^-)_2$ (1), at –40 °C in *a*HF and Acetone-D$_6$ as external solvent.

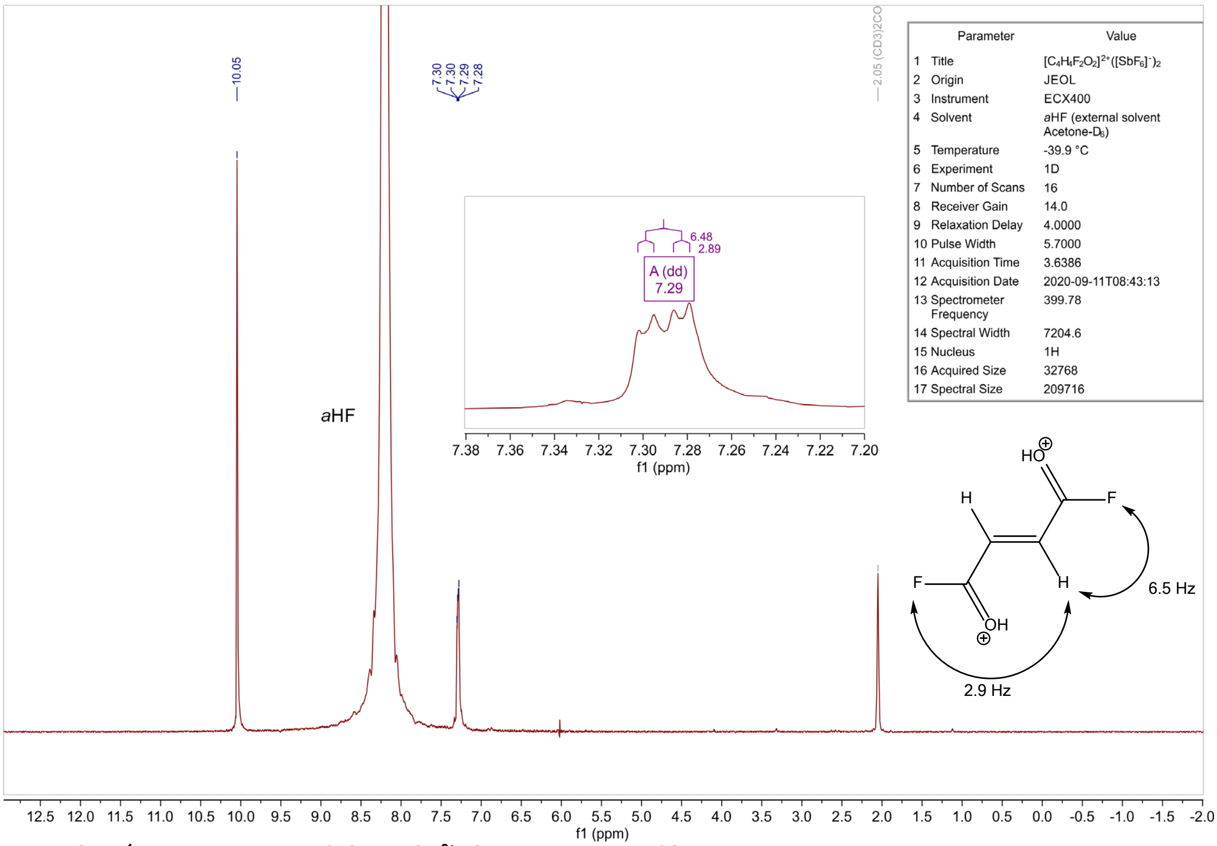

Figure S22: ^{1}H NMR spectrum of $[C_4H_4F_2O_2]^{2+}([SbF_6]^-)_2$ (2), at –40 °C in *a*HF and Acetone-D$_6$ as external solvent.

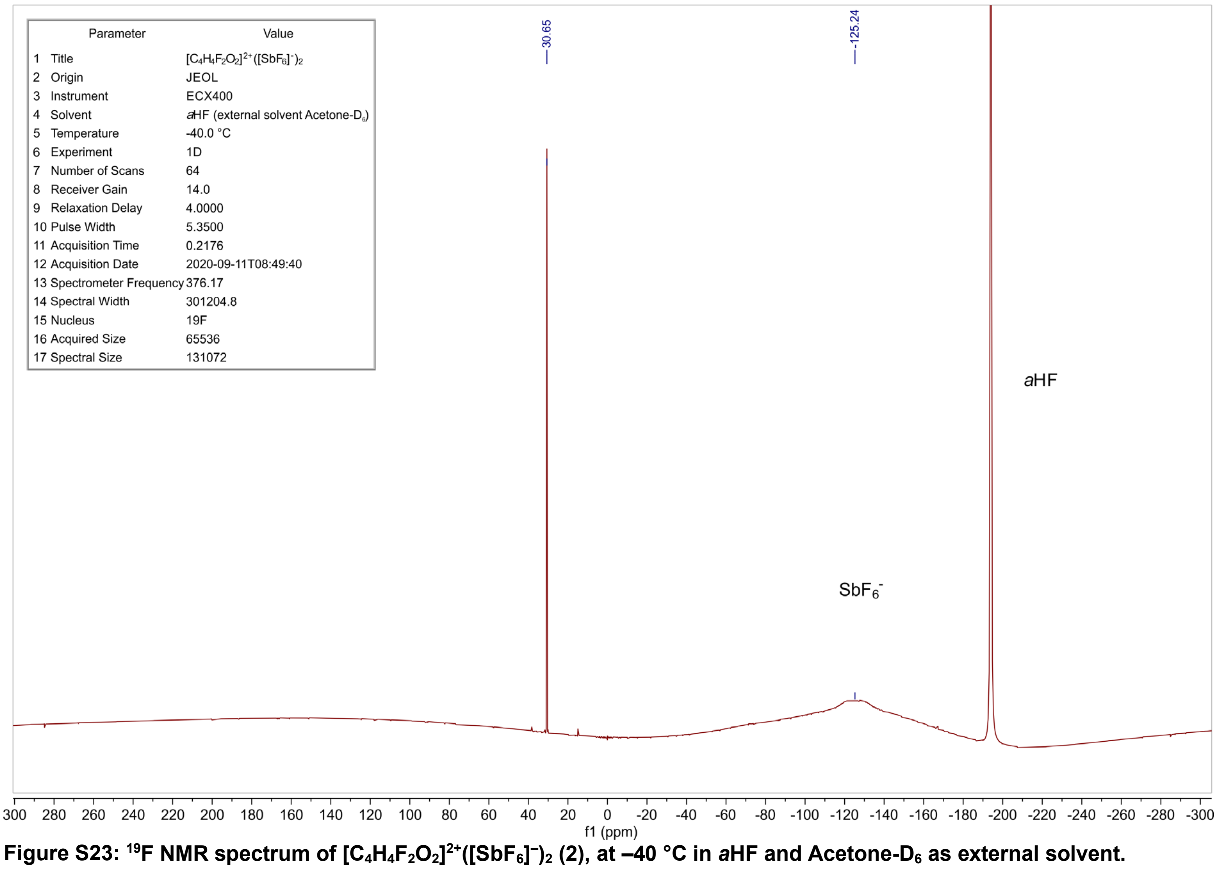

Figure S23: ^{19}F NMR spectrum of $[C_4H_4F_2O_2]^{2+}([SbF_6]^-)_2$ (2), at –40 °C in *a*HF and Acetone-D$_6$ as external solvent.

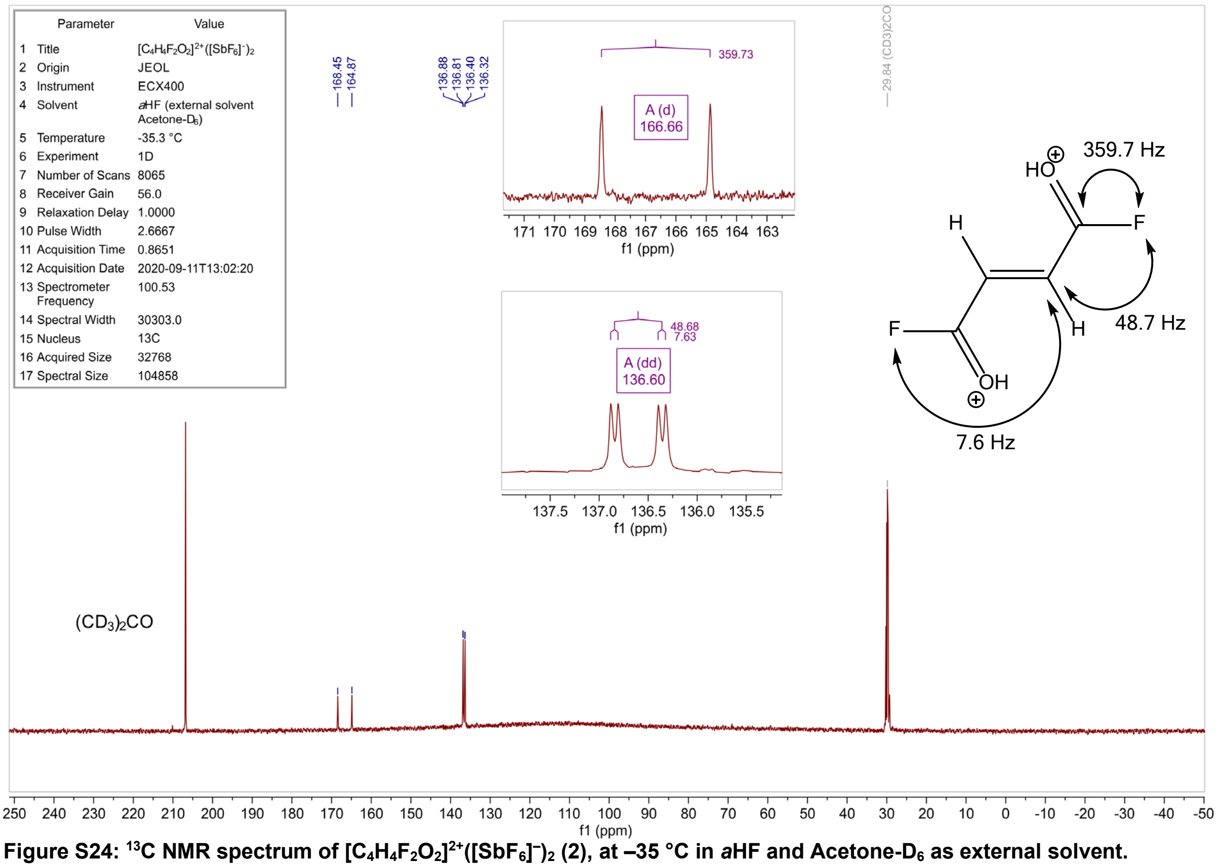

Figure S24: ^{13}C NMR spectrum of $[C_4H_4F_2O_2]^{2+}([SbF_6]^-)_2$ (2), at –35 °C in *a*HF and Acetone-D$_6$ as external solvent.

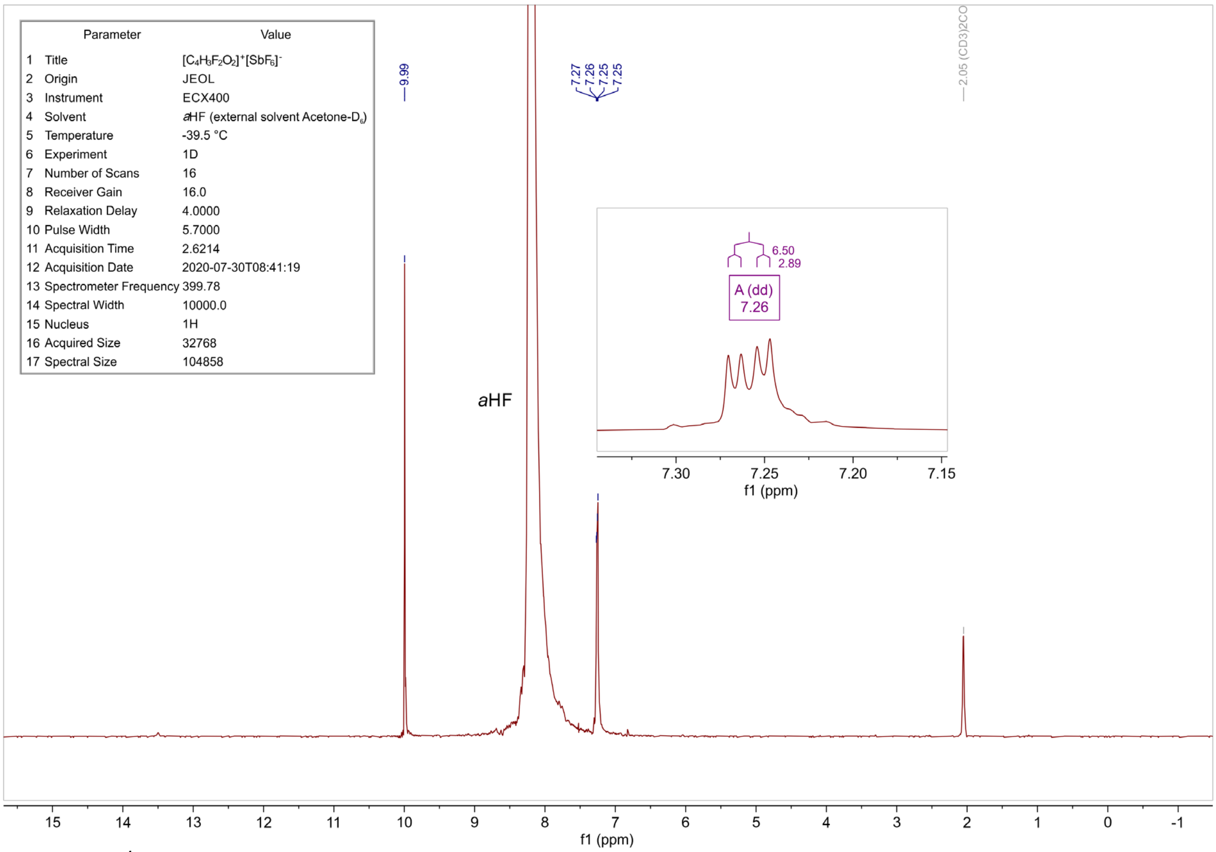

Figure S25: ^{1}H NMR spectrum of $[C_4H_3F_2O_2]^{+}[SbF_6]^{-}$ (4), at –40 °C in *a*HF and Acetone-D_6 as external solvent.

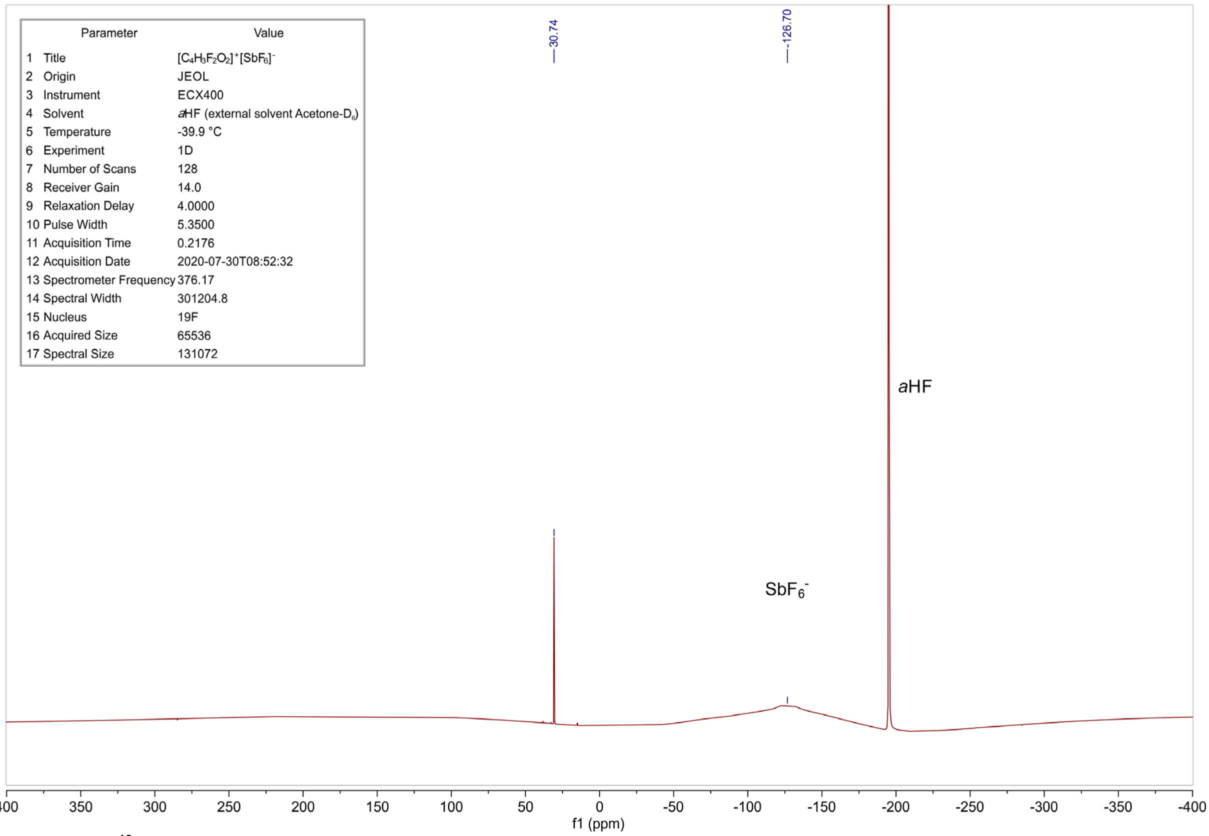

Figure S26: ^{19}F NMR spectrum of $[C_4H_3F_2O_2]^{+}[SbF_6]^{-}$ (4), at –40 °C in *a*HF and Acetone-D_6 as external solvent.

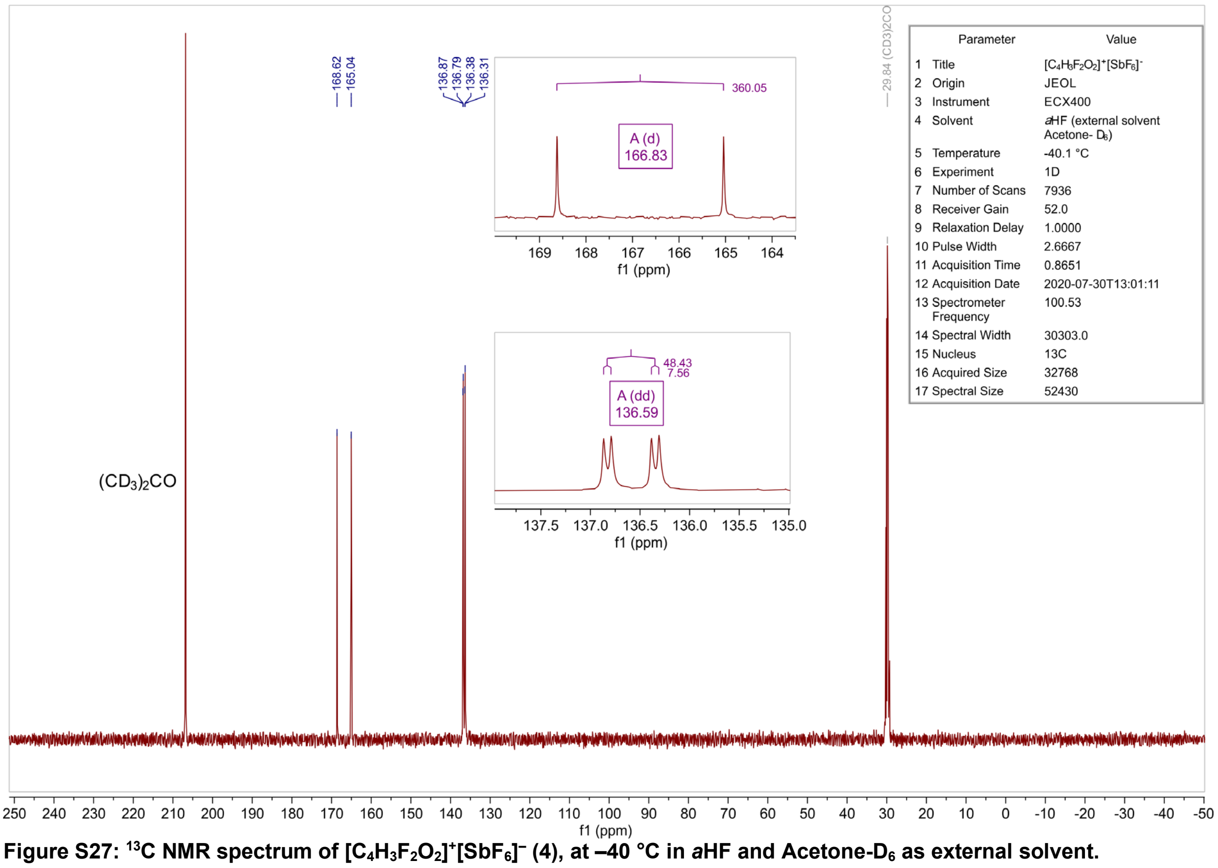

Figure S27: ^{13}C NMR spectrum of $[C_4H_3F_2O_2]^+[SbF_6]^-$ (4), at –40 °C in *a*HF and Acetone-D$_6$ as external solvent.

Table S9: Calculated bond lengths and angles of the free cation $[C_4H_4F_2O_2]^{2+}$ and the HF complex of the cation $[C_4H_4F_2O_2 \cdot 2\ HF]^{2+}$ in comparison with the experimental structural parameters of $[C_4H_4F_2O_2]^{2+}([AsF_6]^-)_2$ (1). The estimated standard deviation is marked in parentheses. Symmetry operations: *i = 1–x, y, 0.5–z*.

	Free cation[a] $[C_4H_4F_2O_2]^{2+}$	HF complex of the cation[a] $[C_4H_4F_2O_2 \cdot 2\ HF]^{2+}$	Crystal structure of $[C_4H_4F_2O_2]^{2+}([AsF_6]^-)_2$ **(1)**	
Bond length [Å]				
C1–F1	1.267	1.278	C1–F1 (C–F)	1.281(2)
C1–O1	1.254	1.240	C1–O1 (C=O)	1.223(2)
C1–C2	1.471	1.471	C1–C2 (C–C)	1.465(2)
C2–C3	1.342	1.339	C2–C2*i* (C=C)	1.330(3)
C3–C4	1.471	1.471		
C4–F2	1.267	1.278		
C4–O2	1.254	1.240		
Bond angle [°]				
O1–C1–F1	120.5	120.6	O1–C1–F1	120.2(2)
O1–C1–C2	122.3	123.4	O1–C1–C2	123.4(2)
F1–C1–C2	117.2	116.0	F1–C1–C2	116.4(2)
C1–C2–C3	121.3	121.2	C1–C2–C2*i*	119.1(2)
O2–C4–F2	120.5	120.6		
O2–C4–C3	122.3	123.4		
F2–C4–C3	117.2	116.0		
C2–C3–C4	121.3	121.2		
Angle of torsion [°]				
O1–C1–C2–C3	0.0	0.0	O1–C1–C2–C2*i*	9.5(2)
F1–C1–C2–C3	–180.0	–180.0	F1–C1–C2–C2*i*	–169.7(1)
O2–C4–C3–C2	0.0	0.0		
F2–C4–C3–C2	–180.0	–180.0		

[a] Calculated at the B3LYP/aug-cc-pVTZ level of theory.

Table S10: Selected experimental vibrational frequencies [cm^{-1}] of $[C_4H_4F_2O_2]^{2+}([AsF_6]^-)_2$ (1) and calculated vibrational frequencies [cm^{-1}] of $[C_4H_4F_2O_2]^{2+}$ and $[C_4H_4F_2O_2 \cdot 2\ HF]^{2+}$.

$[C_4H_4F_2O_2]^{2+}([AsF_6]^-)_2$ **(1)** exp.[a]		$[C_4H_4F_2O_2]^{2+}$ calc.[b]	$[C_4H_4F_2O_2 \cdot 2\ HF]^{2+}$ calc.[b]	Assignment
IR	Raman	IR/Raman	IR/Raman	
3109 vs		3181 (82/0)	3190 (60/0)	ν_{as}(C–H)
	3098 (24)	3179 (0/74)	3188 (0/79)	ν_{s}(C–H)
3182 vs		3494 (1286/0)	2490 (7585/0)	ν_{as}(O–H)
	1717 (49)	1654 (0/222)	1713 (0/8)	ν_{s}(C=O)
	1625 (42)	1696 (0/81)	1685 (0/384)	ν(C=C)
1641 m		1646 (510/0)	1681 (816/0)	ν_{as}(C=O)
1450 m		1486 (1020/0)	1485 (717/0)	ν_{as}(C–F)
	1441 (8)	1470 (0/3)	1454 (0/4)	ν_{s}(C–F)
	1304 (38)	1328 (0/18)	1337 (0/7)	δ_{s}(CCH)
1317 w		1284 (33/0)	1302 (323/0)	δ_{as}(CCH)
	1244 (20)	1140 (0/24)	1280 (0/38)	δ_{s}(COH)
1263 m		1130 (307/0)	1252 (216/0)	δ_{as}(COH)
1153 w		703 (201/0)	1023 (240/0)	γ_{as}(COH)
	946 (24)	987 (0/7)	995 (0/6)	ν_{s}(C–C)
	896 (23)	947 (0/3)	948 (0/4)	γ_{s}(HCCH)
926 w		920 (85/0)	933 (127/0)	ν_{as}(C–C)
	693 (19)	649 (0/9)	712 (0/11)	δ_{s}(COF)
673 m		575 (83/0)	647 (56/0)	δ_{as}(COF)
	608 (8)	593 (0/0)	625 (0/1)	γ_{s}(CCOF)

[a] Abbrevations for IR intensities: v = very, s = strong, m = medium, w = weak. IR intensities in km/mol; Raman intensities in Å^4/u. Experimental Raman activities are relative to a scale of 1 to 100.
[b] Calculated on the B3LYP/aug-cc-pVTZ level of theory.

Table S11: Calculated bond lengths and angles of the free cation $[C_4H_3F_2O_2]^+$ and the HF complex of the cation $[C_4H_3F_2O_2 \cdot HF]^+$ in comparison with the experimental structural parameters of $[C_4H_3F_2O_2]^+[SbF_6]^-$ (4). The estimated standard deviation is marked in parentheses.

	Free cation[a] $[C_4H_3F_2O_2]^+$	HF complex of the cation[a] $[C_4H_3F_2O_2 \cdot HF]^+$	Crystal structure of $[C_4H_3F_2O_2]^+[SbF_6]^-$ **(4)**	
Bond length [Å]				
C2–C3	1.347	1.344	C2–C3	1.334(7)
C1–C2	1.492	1.491	C1–C2	1.476(8)
C3–C4	1.422	1.429	C3–C4	1.454(7)
C1–O1	1.180	1.180	C1–O1	1.187(6)
C4–O2	1.269	1.257	C4–O2	1.239(6)
C1–F1	1.337	1.340	C1–F1	1.332(6)
C4–F2	1.288	1.292	C4–F2	1.285(6)
Bond angle [°]				
O1–C1–F1	123.9	123.5	O1–C1–F1	120.7(5)
O2–C4–F2	117.6	118.0	O2–C4–F2	118.6(4)
O1–C1–C2	123.9	124.2	O1–C1–C2	126.4(5)
O2–C4–C3	121.2	121.7	O2–C4–C3	122.5(5)
F1–C1–C2	112.2	112.2	F1–C1–C2	112.9(4)
F2–C4–C3	121.3	120.2	F2–C4–C3	118.9(4)
C1–C2–C3	123.0	123.1	C1–C2–C3	122.1(5)
C4–C3–C2	120.7	121.0	C4–C3–C2	118.6(5)
Angle of torsion [°]				
O1–C1–C2–C3	179.9	–180.0	O1–C1–C2–C3	–174.5(5)
O2–C4–C3–C2	180.0	–180.0	O2–C4–C3–C2	172.1(5)
F1–C1–C2–C3	–0.1	0.0	F1–C1–C2–C3	7.1(7)
F2–C4–C3–C2	–0.0	0.0	F2–C4–C3–C2	–6.2(7)
C1–C2–C3–C4	–180.0	180.0	C1–C2–C3–C4	–177.1(5)

[a] Calculated at the B3LYP/aug-cc-pVTZ level of theory.

Table S12: Crystal data and structure refinement of $[C_4H_4F_2O_2]^{2+}([AsF_6]^-)_2$ (1), $[C_4H_4F_2O_2]^{2+}([SbF_6]^-)_2$ (2) and $[C_4H_3F_2O_2]^+[SbF_6]^-$ (4).

	$[C_4H_4F_2O_2]^{2+}([AsF_6]^-)_2$ **(1)**	$[C_4H_4F_2O_2]^{2+}([SbF_6]^-)_2$ **(2)**	$[C_4H_3F_2O_2]^+[SbF_6]^-$ **(4)**
Molecular Formula	$C_4H_4As_2F_{14}O_2$	$C_4H_4F_{14}O_2Sb_2$	$C_4H_3F_8O_2Sb$
M_r[g·mol^{-1}]	499.91	593.57	356.82
Crystal size [mm^3]	0.44 x 0.18 x 0.04	0.18 x 0.15 x 0.14	0.309 x 0.261 x 0.136
Crystal system	orthorhombic	monoclinic	monoclinic
Space group	*Pbcn*	$P2_1/c$	*Cc*
a [Å]	9.4255(3)	5.3576(2)	5.5007(5)
b [Å]	7.6954(2)	8.0884(3)	17.9058(13)
c [Å]	17.0276(6)	15.3046(6)	8.9660(6)
α [°]	90	90	90
β [°]	90	97.603(4)	96.007(7)
γ [°]	90	90	90
V [A^3]	1235.06(7)	657.39(4)	878.25(12)
Z	4	2	4
ρ_{calc} [g·cm^{-3}]	2.689	2.999	2.699
μ [mm^{-1}]	5.595	4.282	3.254
$\lambda_{MoK\alpha}$ [Å]	0.71073	0.71073	0.71073
F(000)	944	544	664
T [K]	109(2)	112(2)	293(2)
h, k, l range	–13:14; –11:11; –24:24	–7:7; –12:12; –19:22	–8:8; –27:26; –13:13
Measured reflexes	11992	6711	8944
Unique reflexes	2087	2188	2950
R_{int}	0.0266	0.0342	0.0300
Parameters	103	103	138
R(F)/wR(F^2)[a] (all data)	0.0286/0.0510	0.0432/0.0702	0.0297/0.0556
Weighting scheme[b]	0.021000/0.760500	0.030100	0.027300/0.235600
S (Gof)[c]	1.065	1.087	1.049
Residual density [e·Å^{-3}]	0.555/–0.545	2.978/–0.859	0.763/–0.470
Device	Oxford XCalibur	Oxford XCalibur	Oxford XCalibur
CCDC	2065273	2065274	2065275

[a] $R_1 = \Sigma||F_o|-|F_c||/\Sigma|F_o|$;
[b] $wR_2 = [\Sigma[w(F_o^2-F_c^2)^2]/\Sigma[w(F_o)^2]]^{1/2}$; $w = [\sigma_c^2(F_o^2)+(xP)^2+yP]^{-1}$; $P = (F_o^2+2F_c^2)/3$
[c] GoF = $\{\Sigma[w(F_o^2-F_c^2)^2]/(n-p)\}^{1/2}$ (n = number of reflexions; p = total number of parameters).

Table S13: Cartesian coordinates of calculated minimum structures of $[C_4H_4F_2O_2 \cdot 2\ HF]^{2+}$ at the B3LYP/aug-cc-pVTZ level of theory.

Atom	x	y	z
F	0.000013377	–0.000000413	0.000042976
F	–0.000003126	–0.000004837	–0.000004708
O	–0.000029351	–0.000015410	–0.000039845
C	0.000025460	0.000015901	0.000022157
C	0.000021739	–0.000000957	0.000003606
H	–0.000015723	–0.000000449	–0.000007002
H	0.000009037	0.000003322	0.000022123
F	–0.000000030	0.000005764	0.000005868
O	0.000036465	0.000017531	0.000000629
C	–0.000034905	–0.000004712	–0.000032434
C	–0.000020536	–0.000005824	0.000001747
H	0.000015001	0.000000877	0.000006768
H	0.000007038	–0.000017021	0.000027575
F	0.000001120	0.000005147	–0.000010942
H	–0.000024590	0.000002658	–0.000037630
H	–0.000000977	–0.000001577	–0.000000888

Table S14: Cartesian coordinates of calculated minimum structures of $[C_4H_4F_2O_2]^{2+}$ at the B3LYP/aug-cc-pVTZ level of theory.

Atom	x	y	z
F	0.000008196	0.000000586	–0.000022814
O	–0.000015896	0.000001265	0.000199621
C	0.000055132	0.000000972	0.000060027
C	0.000282106	–0.000005391	–0.000002706
H	–0.000195442	0.000005197	–0.000071303
H	–0.000024940	–0.000002629	–0.000228923
F	–0.000008196	0.000000586	0.000022814
O	0.000015896	0.000001265	–0.000199621
C	–0.000055132	0.000000972	–0.000060027
C	–0.000282106	–0.000005391	0.000002706
H	0.000195442	0.000005197	0.000071303
H	0.000024940	–0.000002629	0.000228923

Table S15: Cartesian coordinates of calculated minimum structures of $[C_4H_2D_2F_2O_2 \cdot 2\ HF]^{2+}$ at the B3LYP/aug-cc-pVTZ level of theory.

Atom	x	y	z
F	0.000013507	0.000017541	0.000002734
F	–0.000001060	0.000002156	–0.000000597
O	–0.000009266	–0.000012576	0.000007513
C	0.000007268	0.000007375	0.000006902
C	0.000001417	0.000008364	–0.000004810
H	–0.000001451	–0.000005016	–0.000000170
D	0.000000848	0.000006839	–0.000012522
F	0.000001605	–0.000001406	–0.000001038
O	0.000003976	0.000017293	–0.000008303
C	–0.000012506	–0.000015302	0.000001512
C	0.000000882	–0.000007256	0.000002060
H	0.000001266	0.000004649	–0.000000600
D	0.000001035	–0.000005295	0.000008761
F	0.000002482	–0.000000823	–0.000002388
H	–0.000009827	–0.000013328	0.000000627
H	–0.000000177	–0.000003216	0.000000318

Table S16: Cartesian coordinates of calculated minimum structures of $[C_4H_3F_2O_2 \cdot HF]^+$ at the B3LYP/aug-cc-pVTZ level of theory.

Atom	x	y	z
F	–0.000004615	–0.000026454	0.000062575
F	0.000002273	0.000002094	0.000002459
F	0.000000584	–0.000001197	–0.000001043
O	0.000000556	–0.000029763	0.000123924
H	–0.000000662	0.000051268	–0.000194542
O	0.000002181	–0.000000945	0.000002396
C	0.000000366	–0.000007563	–0.000007871
C	0.000002634	0.000000807	0.000002265
C	–0.000003282	–0.000002258	–0.000003301
H	0.000004871	–0.000000044	0.000000677
C	0.000001780	0.000004404	0.000006324
H	–0.000002177	0.000002173	–0.000001037
H	–0.000004509	0.000007477	0.000007172

Table S17: Cartesian coordinates of calculated minimum structures of $[C_4H_3F_2O_2]^+$ at the B3LYP/aug-cc-pVTZ level of theory.

Atom	x	y	z
C	0.000001589	0.000011539	0.000020116
H	0.000006770	0.000000449	–0.000002211
C	0.000002711	0.000000591	–0.000057211
H	0.000002476	0.000005970	0.000022150
C	0.000008598	–0.000032851	–0.000008687
C	0.000042868	–0.000076509	0.000010641
O	–0.000013294	0.000029042	0.000002599
F	–0.000009391	–0.000000637	0.000003279
F	–0.000030296	0.000058047	0.000004921
O	–0.000006734	0.000017732	0.000004546
H	–0.000005297	–0.000013371	–0.000000142

[1] M. C. Bayer, C. Jessen, A. J. Kornath, *Z. Anorg. Allg. Chem.* **2021**, *647*, 258.
